# Mammalian lipids: structure, synthesis and function

**DOI:** 10.1042/EBC20200067

**Published:** 2021-11-02

**Authors:** Shamshad Cockcroft

**Affiliations:** Department of Neuroscience, Physiology and Pharmacology, Division of Biosciences, University College London, 21 University Street, London WC1E 6JJ, U.K.

**Keywords:** phospholipases, Cholesterol, phosphatidylinositol, sphingolipids, Signalling

## Abstract

Lipids are essential constituents of cellular membranes. Once regarded merely as structural components, lipids have taken centre stage with the discovery of their roles in cell signalling and in the generation of bioactive metabolites. Lipids regulate many physiological functions of cells and alterations in membrane lipid metabolism are associated with major diseases including cancer, Type II diabetes, cardiovascular disease and immune disorders. Understanding lipid diversity, their synthesis and metabolism to generate signalling molecules will provide insight into the fundamental function of the cell. This review summarises the biosynthesis of the lipids of the mammalian cell; phospholipids, sphingolipids and cholesterol and how lipid diversity is achieved. The fatty acids (FAs) are the main building blocks of lipids and contribute to the diversity. Lipid synthesis is intimately connected to their transport within cells; the contribution by proteins that transport lipids, lipid transport proteins will be described. Cellular lipids are metabolised by phospholipases, lipid kinases and phosphatases to make new bioactive metabolites. These transient bioactive metabolites allow cells to respond to the external environment to maintain cellular health. The function of individual metabolites is also highlighted. Bioactive metabolites can be second messengers, or released to the external medium to regulate other cells. Alternatively, bioactive lipids also provide a platform for reversible recruitment of proteins to membranes using their lipid-binding domains. The wide range of physiological processes in which a specific involvement of lipids has been identified explains the need for lipid diversity present in mammalian cells.

## Introduction

Lipids play essential functions in cellular physiology and pathology. They are the structural components of cell membranes, without which cells (and life) would not exist. In addition to this most basic function, lipids serve as energy stores, sources of signalling molecules and platforms for protein recruitment. Lipids are a structurally diverse group of molecules and can be best defined as substances that are soluble in non-polar organic solvents (e.g. chloroform) but not in water. Lipids can be hydrophobic (water-hating) or amphiphilic. An amphiphilic lipid has properties that permit formation of cellular membranes. A hydrophilic headgroup at one end and a hydrophobic region at the other end allows the lipids to line up so that the hydrophobic and hydrophilic regions align with each other to form a monolayer. Two monolayers come together to form the membrane, with the water-soluble hydrophilic portion facing the aqueous environment and the hydrophobic regions facing each other. The plasma membrane of cells separates the inside of a cell from the extracellular space, whilst intracellular membranes form organelles that can segregate different metabolic functions. The main organelles of mammalian cells include the endoplasmic reticulum (ER), the Golgi complex, the mitochondria, endosomes, lysosomes, peroxisomes and secretory vesicles and granules. The lipid composition of the different organelles is distinct reflecting their function.

Most lipids are synthesised at the ER, with some contribution from the Golgi and mitochondria. The ER is the organelle with the largest membrane area, organised into a network of branching tubules and flattened sacs that extends throughout the cytosol. The membranes of the tubules and the flattened sacs are continuous with the nuclear membrane. The ER and nuclear membranes enclose a single internal space, the luminal space. The ER makes close contact with most of the organelles including mitochondria, endosomes, plasma membranes and lysosomes. These regions of close contact are known as membrane contact sites and enable lipid exchange between the organelles. The distance between the two membranes is in the region of ∼30 nm, sufficiently close that a protein can tether the membranes. As lipid metabolism can be spread between different membrane compartments, proteins that bind and transport lipids are required. These proteins are known as lipid transfer proteins (LTPs). LTPs often are large multidomain proteins with the lipid transfer domain present together with other domains which function to bring the two membrane compartments close together. However, some LTPs comprise a lipid transfer domain only and rely on other tether proteins to bring the membranes together. The lipid transfer domain of LTPs is highly specific; often it can bind two different lipids allowing for lipid exchange to take place.

Another important aspect of lipid biosynthesis is the side (leaflet) of the bilayer that the lipid is synthesised. Lipid synthesis often requires the movement of a lipid from one leaflet to the other. In intracellular organelles such as the ER, one leaflet of the lipid bilayer faces the cytosol and therefore accessible to lipid transport proteins whilst the other leaflet is exposed to the lumen. Therefore, if a lipid is partially synthesised on the cytosolic-facing leaflet and needs to be transferred to the luminal leaflet where the rest of the biosynthetic machinery is localised, the lipids need to be flipped across the bilayer. Three categories of interleaflet lipid transporters are identified: scramblases, flippases and floppases. Scramblases transport lipids between the two leaflets in both directions. This is a non-selective process and does not require ATP. In contrast, flippases and floppases transport specific lipids between the two leaflets and use ATP as an energy source. Flippases transfer lipids from the luminal leaflet to the cytoplasmic leaflet, whilst floppases transfer in the opposite direction. (note that the luminal leaflet is the equivalent of the leaflet facing the extracellular space). In this review, the structure, the synthesis and the function of lipids will be described. Lipids display large structural complexity, with ∼40000 different lipids identified to date (http://www.lipidmaps.org). Many lipids present in plants, fungi, archaea and bacteria are not made in mammalian cells and will not be considered here.

## Lipids of mammalian cells

**Figure 1:**
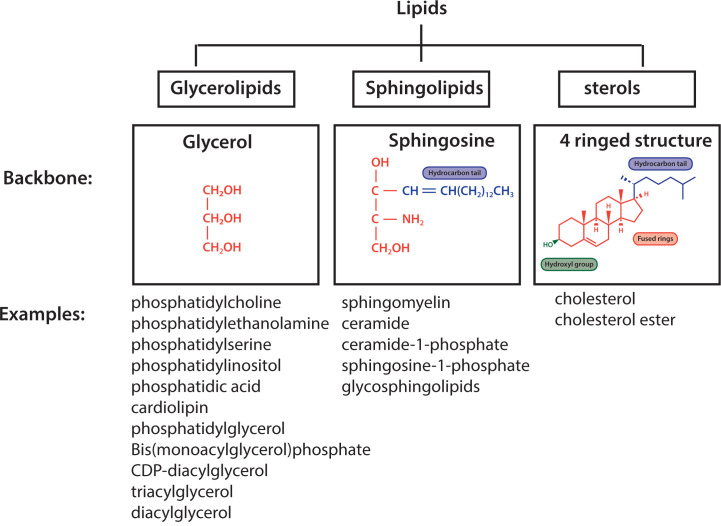
Three classes of lipids of mammalian cell Glycerolipids use glycerol as their backbone, sphingolipids use a sphingoid backbone and sterols are a four-ringed structure with a hydrophobic tail and a hydroxyl group at opposite ends. Examples of lipids from the individual classes present in mammalian cells are provided.

The main lipids present in a biological membrane of a mammalian cell can be subdivided into three classes: glycerolipids, sphingolipids and sterols (Figure 1). Glycerolipids use glycerol as their backbone whilst sphingolipids use sphingosine, an amino alcohol. The sphingoid backbone encompasses a hydrophobic tail (Figure 1). In contrast with glycerolipids and sphingolipids, sterols have four rings arranged in a specific molecular configuration with a hydroxyl group at one end and a short hydrocarbon side-chain at the opposite end (Figure 1).

**Figure 2:**
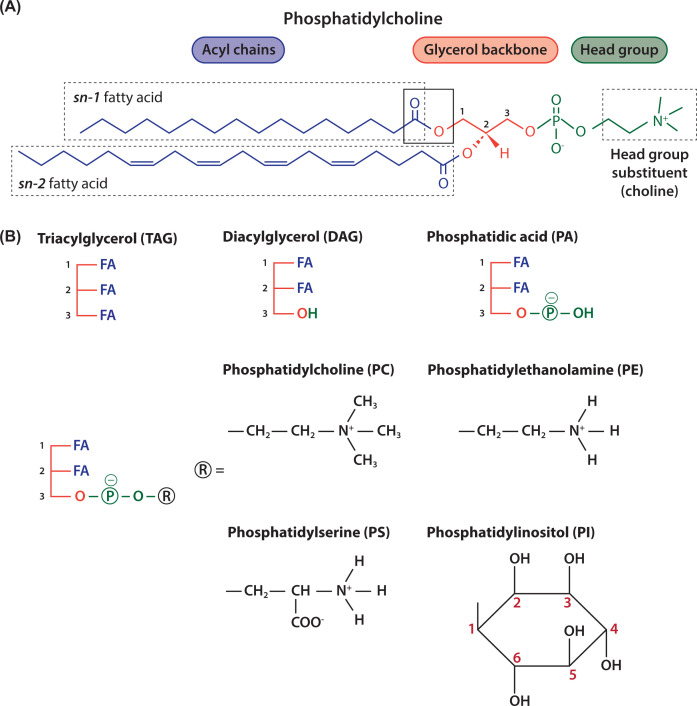
Schematic representation of glycerolipids (**A**) Phosphoglycerides have a glycerol backbone (coloured red) with FAs at the *sn-1* and *sn-2* position (coloured blue) and a phosphate moiety that links to a headgroup at *sn-3* position (coloured green). In this example, the headgroup is choline and therefore the phospholipid is phosphatidylcholine (PC) (sn, stereochemical numbering). (**B**) The glycerol backbone with three FAs is called TAG. The glycerol backbone with two FAs is diacylglycerol (DAG). Addition of a phosphate to DAG makes phosphatidic acid (PA), the simplest phospholipid. Different headgroups (R) can be attached to the phosphate; addition of choline makes PC, ethanolamine makes phosphatidylethanolamine (PE), serine makes phosphatidylserine (PS) and inositol makes phosphatidylinositol (PI).

Lipids used for energy storage are the glycerolipid, triacylglycerol (TAG), where each of the three hydroxyls of the glycerol backbone have fatty acids (FAs) attached to them (Figure 2). Lipids that contain a phosphate are known as phospholipids and these are the main lipids of the membrane together with cholesterol (Figure 2).

## Structure of phospholipids

Glycerophospholipids, comprise a glycerol backbone, two FAs a phosphate moiety and a headgroup that gives the phospholipid its name (Figure 2A). The three carbons of glycerol are labelled as *sn-*1, *sn-*2 and *sn-*3. (*sn* stands for stereochemical numbering). Two of the three hydroxyls of glycerol (*sn-*1 and *sn-*2) are esterified with FAs which makes diacylglycerol (DAG) and addition of a phosphate to the third hydroxyl of glycerol (*sn-*3) makes phosphatidic acid (PA) (Figure 2B). PA is the simplest phospholipid and serves as a primary building block to make other phospholipids (Figure 5).

**Figure 3:**
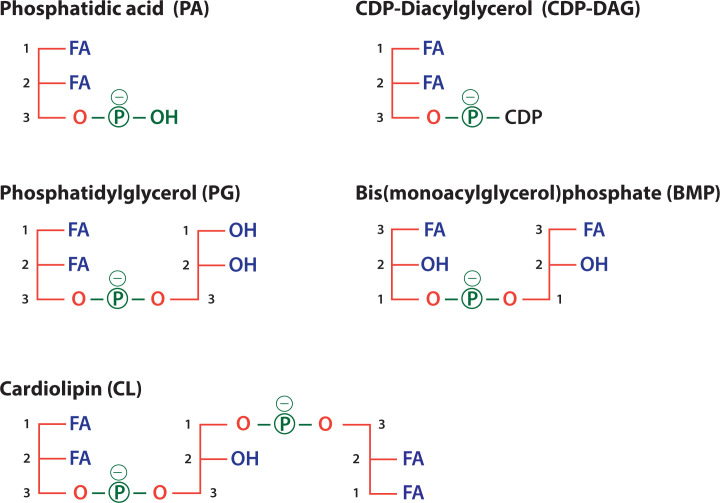
Schematic representations of PA, PG, CL, CDP-diacylglycerol and bis(monoacylglycero)phosphate PA is the precursor for the synthesis of CDP-DAG, PG, CL and BMP. PG and BMP are both based on two glycerol molecules attached by a phosphate moiety and two FAs but with different stereochemistry. CL (alternative name: diphosphatidylglygerol) is synthesised from PG and CDP-DAG. Abbreviation: CDP-DAG, CDP-diacylglycerol.

Addition of different headgroups to the phosphate of PA generates the diverse phospholipids (Figure 2B and Table 1). Thus, addition of the headgroup choline makes phosphatidylcholine (PC), the most abundant phospholipid present in all membranes. Other headgroups are ethanolamine to make phosphatidylethanolamine (PE), serine to make phosphatidylserine (PS) and inositol to make phosphatidylinositol (PI) (Figure 2B). Addition of glycerol as a headgroup to PA produces phosphatidylglycerol (PG) which can then be converted into cardiolipin (CL) (Figure 3). CL, a phospholipid, so named as it was originally identified as a major component of heart lipids (CL is also known as diphosphatidylglycerol). It is unusual as it has four acyl chains (Figure 3). Essentially, two PA molecules are attached to the *sn-1* and *sn-*3 positions of a glycerol backbone to make CL.

**Table 1:** Phospholipids present in membranes of mammalian cells

Phospholipids	Abbreviations	Comments
Major phospholipids		
Phosphatidylcholine (45–55%)	PC (PtdCho)	Major phospholipid of the cells
Phosphatidylethanolamine (15–25%)	PE (PtdEtn)	Major phospholipid of the cells after PC
Phosphatidylserine (10–15%)	PS (PtdSer)	Mainly localised to the cytosolic leaflet of the plasma membrane
Sphingomyelin (5–10%)	SM	Mainly localised to the outer leaflet of the plasma membrane
Phosphatidylinositol (5–10%)	PI (PtdIns)	Inositol headgroup can be phosphorylated at three positions to make seven phosphorylated PIs (Figure 6)
Cardiolipin (also known as diphosphatidylglycerol) (2–5%)	CL	Synthesised in mitochondria
Minor phospholipids		
Phosphatidic acid	PA (PtdOH)	Intermediate in lipid synthesis; signalling lipid
CDP-diacylglycerol (alt. CMP-phosphatidate)	CDP-DAG (CMP-PA)	Metabolic intermediate for PI, PG and CL biosynthesis
Phosphatidylglycerol	PG (PtdGro)	Synthesised in mitochondria
bis(monoacylglycerol)phosphate (also known as lysobis phosphatidic acid)	BMP (LBPA)	Late endosomes and lysosomes

The phospholipid composition of a typical cell is indicated. The percentages of the major phospholipids is indicated in parenthesis. The minor lipids are present between 0.5 and 2%. All the phospholipids have a glycerol backbone except sphingomyelin which contains a sphingoid backbone. Two systems are commonly used for the abbreviation of phospholipids. The simpler system is used here such that phosphatidylcholine is abbreviated to PC, not PtdCho. The abbreviations for the other system are provided in parenthesis.

Two other phospholipids which are present at very low concentrations are CDP-diacylglycerol (CDP-DAG) and bis(monoacylglycerol)phosphate (BMP) (Figure 3). CDP-DAG is a metabolic intermediate in the synthesis of PI at the ER (Figure 5) and of PG and CL in mitochondria (Figure 8). Compared with these phospholipids, BMP is unique in its stereochemical configuration. In most phospholipids, the phosphate is attached at the *sn-3* position but in BMP, the phosphate is at *sn-*1 (Figure 3). BMP is specifically localised in the late endosomes and lysosomes. This stereochemistry protects it from degradation by lysosomal enzymes.

### FAs as building blocks for lipids

**Figure 4:**
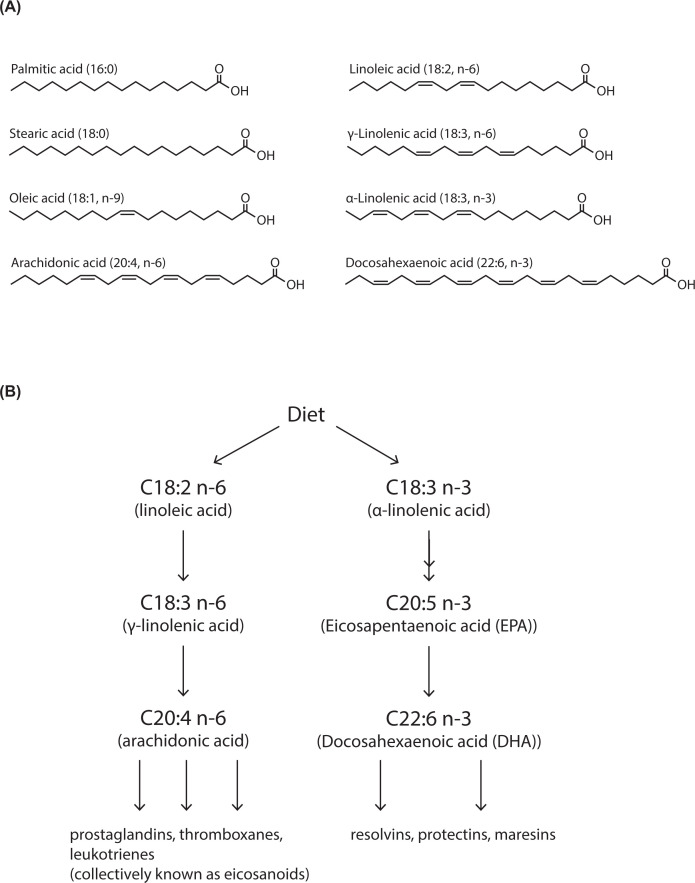
FA diversity due to chain length and degree of unsaturation (**A**) FAs comprise a carbon acyl chain with a carboxylic acid at one end. FAs are defined by their acyl chain length and by the number of double bonds. Thus, oleic acid is (C18:1 n-9). C18 denotes the length of the carbon chain and the number of double bonds is indicated after the colon. The position of the first double bond is denoted by *n* (number) where the carbon chain is counted from the hydrocarbon end. (**B**) Linoleic acid (C18:2 n-6) and α-linolenic acid C18:3 n−3) are essential FAs and are acquired from the diet. They can be further elongated by addition of acetyl groups by elongase enzymes and double bonds added by desaturase enzymes. n-3 and n-6 FAs are also known as ω3 and ω6 FAs, respectively. See Box 1 for explanation for the nomenclatures used for the position of the double bonds.

Phospholipid diversity is not only due to different headgroups but also as a result of the acyl chains of the fFAs FAs are the fundamental building blocks not only of phospholipids, but also sphingolipids and TAG. In addition, cholesterol has to be esterified with FAs for storage. FAs comprise a carboxylic acid with a hydrocarbon acyl chain which can vary in both length (C_14_–C_26_) and degree of saturation (number of double bonds) (Figure 4A and Box 1). The acyl chains with no double bonds are referred to as saturated FAs. FAs can contain a single double bond (monounsaturated) or multiple double bonds (polyunsaturated).

Box 1 **FAs: representation of carbon length and position of double bonds**

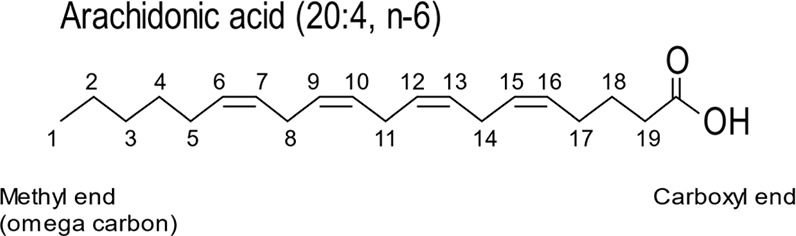

FAs are characterised by chain length of carbon atoms and number of double bonds. For chain length, number of carbons is represented by ‘C’ and the number after the colon refers to the number of double bonds. Therefore, arachidonic acid is C20:4 is a FA of 20 carbons in length with four double bonds. Two systems are in use to denote the position of the double bond. One system counts from the methyl end and uses ‘*n’* (in dietary literature, the carbon at the methyl end is referred to as omega (ω) carbon). Therefore, a double bond between carbons 6 and 7 in arachidonic acid is denoted as C20:4, n-6 (or ω6). Only the first double bond is indicated in this nomenclature. The second system is used for desaturase enzymes that introduce double bonds and is counted from the carboxyl carbon (referred as Δn). Thus Δ9-desaturase introduces a double bond between carbons 9 and 10 and Δ15-desaturase introduces a double bond between carbons 15 and 16.

The most common saturated FAs are palmitic acid and stearic acid found in many phospholipids. Palmitic acid contains 16 carbons and is referred to as C16:0 (in this nomenclature, 16 denotes the number of carbons in the chain, whereas 0 indicates the number of double bonds). Stearic acid contains 18 carbons (C18:0). The most common monounsaturated FA is oleic acid, which is 18 carbons in length with 1 double bond (C18:1 n-9). n-9 refers to the location of the double bond, counting the carbon from the terminal methyl group (Figure 4A). The double bond is normally in the *cis* configuration, introducing a kink into the molecular shape. Double bonds also restrict the motion of the acyl chain. The most common polyunsaturated FAs are linoleic acid (C18:2 n-6), γ-linolenic acid (C18:3 n-6), α-linolenic acid (C18:3 n-3) and arachidonic acid (C20:4 n-6) (Figure 4A).

### FA synthesis

Humans can synthesise many of the FAs required for lipid synthesis with some exceptions and are reliant on the food consumed. Linoleic acid (C18:2 n-6) and α-linolenic acid (C18:3 n-3) are essential FAs which can be further metabolised to make longer FAs by elongase enzymes and with more double bonds by desaturase enzymes (Figure 4B). These derivatives such as arachidonic acid (C20:4 n-6) and docosahexaenoic acid (DHA; C22:6 n-3) are used to make other bioactive metabolites such as prostaglandins. To understand why these particular FAs need to be acquired from the diet, the biosynthetic pathway for FA synthesis needs to be understood.

FA synthesis is initiated by the condensation of malonyl-CoA with acetyl CoA (malonyl CoA is made from acetyl CoA by carboxylation). Addition of acetyl CoA units elongates the chain by two carbons and takes place in the cytosol through a complex of enzymes known as the FA synthase. The maximal chain length achieved by FA synthase is 18 carbons (C18:0). Further elongation of the chain up to C_26_ is achieved through enzymes that are present in the ER. The introduction of double bonds into the acyl chains and thus their desaturation is performed by the enzyme, stearoyl-CoA desaturase 1 (SCD1). SCD1 is also known Δ9 desaturase because it creates a double bond between the 9^th^ and 10^th^ carbon counting from the carboxyl end (see Box 1). SCD1 can convert C16:0 and C18:0 into C16:1 n-7 and C18:1 n-9 respectively. Addition of a second double bond can occur by other desaturase enzymes but only between an existing double bond and the carboxyl group. Four desaturases are present in humans, Δ9, Δ6, Δ5 and Δ4 desaturases. Δ6 and Δ5 desaturases are required for the synthesis of highly unsaturated FAs such as eicosapentaenoic acid (EPA) and DHA synthesised from α-linolenic acid (Figure 4B).

Mammals lack the desaturases to convert oleic acid (C18:1 n-9) into linoleic acid (C18:2 n-6) and α-linolenic acid (C18:3 n-3). Thus, linoleic acid (C18:2 n-6) and α-linolenic acid (C18:3 n-3) are essential dietary FAs (Figure 4B). γ-linolenic acid (C18:3 n-6) can be made from linoleic acid (C18:2 n-6). Longer chain FAs are made from C18:2 n-6 and C18:3 n-3 by elongase and desaturase enzymes (Figure 4B). The three most important polyunsaturated FAs are arachidonic acid, (C20:4 n-6) derived from linoleic acid (C18:2 n-6) and DHA (C22:6 n-3) and EPA (C20:5 n-3) derived from α-linolenic acid (C18:3 n-3) (Figure 4B). These FAs are metabolised by a number of enzymes giving rise to physiologically active metabolites that can activate cells by binding to specific cell surface receptors. In general, arachidonic acid-derived metabolites are pro-inflammatory whilst DHA/EPA-derived metabolites are non- or anti-inflammatory. Arachidonic acid is converted into prostaglandins by the cyclooxygenase enzyme which is the target for aspirin, the most used anti-inflammatory drug in medicine today. The EPA/DHA-derived metabolites are important for resolving inflammation. Thus, the balance of linoleic acid (18:2 n-6) and α-linolenic acid (18:3 n-3) in the diet have important physiological consequences.

### Biosynthesis of phospholipids at the ER

**Figure 5:**
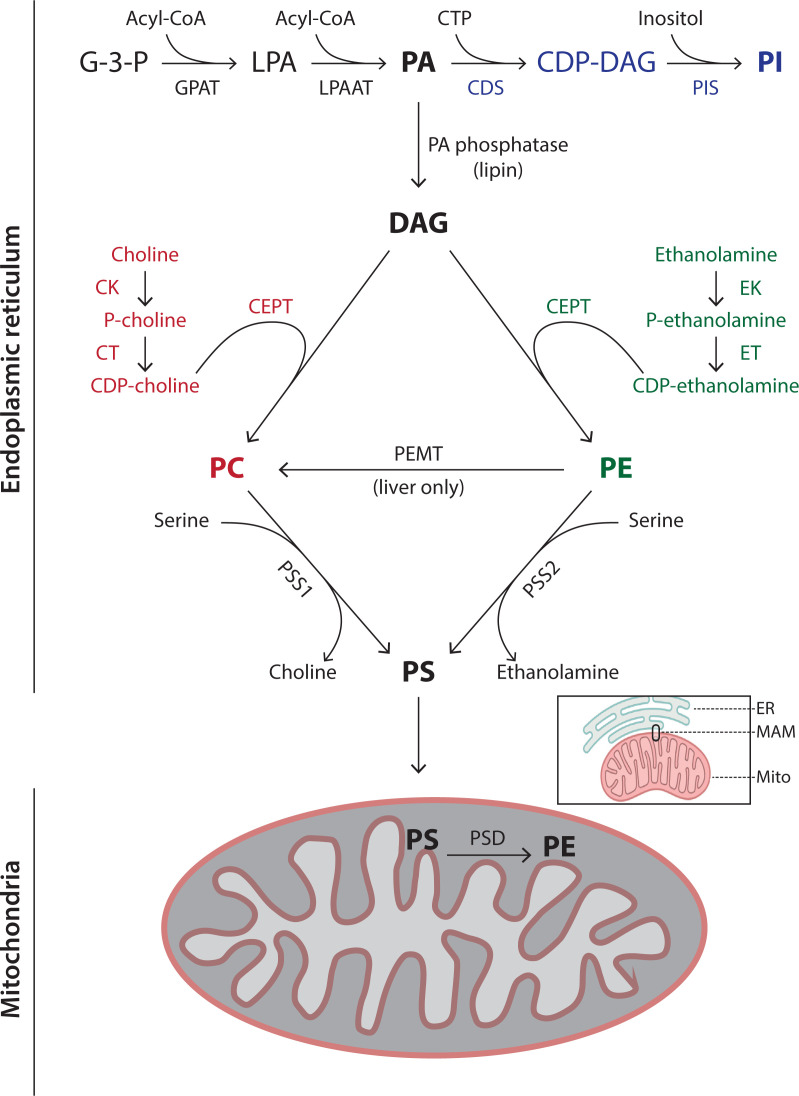
Synthesis of the phospholipids, PC, PE, PS and PI Synthesis of the phospholipids occurs mainly at the ER. PE is additionally synthesised in mitochondria after transfer of PS from the ER through a membrane contact site, called mitochondrial-associated ER membranes (MAMs – see inset) (see text for further details). The names of the enzymes are provided in Table 2.

PA is the precursor of most phospholipids in mammals. Figure 5 outlines the pathways for the synthesis of PI, PC, PE and PS which mainly takes place at the ER with some PE synthesis taking place in mitochondria. The full name of the enzymes involved in the synthesis of the phospholipids are listed in Table 2. The first step in PA synthesis is the sequential acylation of glycerol-3-phosphate (G-3-P) by *glycerol 3-phosphate acyl-transferase* (GPAT). The first acylation occurs at the *sn-*1 position to make lyso-PA (LPA) and the second acylation at the *sn-*2 position by *lyso-PA acyl transferase* (LPAAT). Both enzymes are integral membrane proteins and multiple isoforms are present. For the synthesis of PC, PE and PS, PA is converted into DAG by *PA phosphatases* (also known as *lipins*).

**Table 2:** The enzymes involved in the synthesis of phospholipids

Enzymes involved in phospholipid synthesis	Abbreviations of enzymes	Comments
Acyl-CoA glycerol-3-phosphate acyltransferase	GPAT (4)	Integral membrane proteins; two enzymes are in the ER and two are located in mitochondria
Lyso-phosphatidic acid acyl transferase	LPAAT (3)	Integral membrane proteins at the ER
CDP-diacylglycerol synthase	CDS1 and CDS2	Integral membrane proteins located at the ER
PI synthase (CDP-diacylglycerol:inositol-3-phosphatidyltransferase)	PIS	Integral membrane protein located at the ER
PA phosphatase (Lipin)	PAP (3)	Soluble proteins that transiently associate with the ER
Choline kinase	CKα and CKβ	Soluble proteins; rate-limiting enzyme
CTP-phosphocholine cytidylyltransferase	CTα and CTβ	Soluble proteins that associates with ER membranes in a regulated manner
CDP-choline:1,2 diacylglycerol cholinephosphotransferase	CPT	CPT, an integral enzyme, localises to the Golgi
Ethanolamine kinase	EK1 and EK2	Soluble proteins
CTP:phosphoethanolamine cytidlyltransferase	ET	Soluble protein and does not require membrane association to be active
CDP-ethanolamine: 1,2 diacylglycerol ethanolaminephosphotransferase	CEPT	CEPT, an integral enzyme localised at the ER, can use either CDP-ethanolamine or CDP-choline as substrate
Phosphatidylserine synthase 1 and 2	PSS1 and PSS2	PSS1 and PSS2 replaces the headgroup of PC and PE with serine respectively; both are integral membrane enzymes. Mice knockouts of PSS1 or PSS2 individually are viable but double knockouts are not
Phosphatidylethanolamine N-methyl transferase	PEMT	Enzyme restricted to the liver
Phosphatidylserine decarboxylase	PSD	PSD is localised in the external side of the inner mitochondrial membrane

Some of the enzymes have multiple versions indicated in brackets and can be differentially localised.

#### PC synthesis

PC is present in mammalian cells at ∼45–55% of all phospholipids and is the most abundant phospholipid. The major pathway for PC synthesis occurs via the CDP-choline pathway (Kennedy pathway) (Figure 5, **coloured in red**). First choline is phosphorylated to phosphocholine by *choline kinase* (CK). Next, the enzyme *CTP:phosphocholine cytidylyltransferase* (CT) catalyses the condensation of phosphocholine and CTP thus producing CDP-choline. Finally, *CDP-choline:diacylglycerol choline phosphotransferase* (CEPT) converts CDP-choline and DAG into PC.

PC can also be synthesised from PE in three sequential methylation reactions catalysed by the enzyme, *phosphatidylethanolamine N-methyltransferase* (PEMT). The methyl donor is S-adenosylmethionine (SAM). This pathway is restricted to the liver where 30% of PC is derived through this pathway.

#### PE synthesis

Mammalian cells synthesise PE via two alternative pathways. One pathway is the CDP-ethanolamine pathway (Figure 5, **coloured in green**), analogous to that of PC. Ethanolamine is phosphorylated by *ethanolamine kinase* (EK) and phosphoethanolamine and CTP combine to make CDP-ethanolamine catalysed by *CTP:phosphoethanolamine cytidyltransferase* (ET). Finally, CEPT catalyses the conversion of CDP-ethanolamine and DAG into PE, the same enzyme used for PC synthesis. The second pathway takes place in mitochondria where PS is converted into PE. Both pathways are essential; this is due to the location of the synthesised PE. PE synthesised at the ER cannot get into mitochondria where it is essential for function.

#### PS synthesis

In mammals, PS synthesis takes place at the ER by headgroup exchange from PC or PE by the enzymes, *phosphatidylserine synthase 1* and *2* (PSS1 and PSS2). PSS1 replaces the headgroup of PC with serine and PSS2 catalyses the analogous reaction with PE. The synthesis of PS takes place in a subcompartment of the ER that is associated with mitochondria, called mitochondria-associated membranes (MAMs) (Figure 5, **see inset**) (this subcompartment is now known as a membrane contact site.). For PE synthesis, PS is transported to the mitochondria where it is decarboxylated by *phosphatidylserine decarboxylase* (PSD). PSD is localised to outer surface of the inner mitochondrial membrane (IMM). PS is transferred through the inner mitochondrial space by a lipid transport protein of the PRELID family (another PRELID family member transfers PA for CL synthesis (Figure 8)).

#### Synthesis of PI and its phosphorylated derivatives

The synthesis of PI from PA occurs in two steps. The first step is the conversion of PA and CTP into the lipid intermediate, CDP-DAG by the enzyme, *CDP-diacylglycerol synthase* (CDS). The second step requires inositol where the enzyme, *PI synthase* (PIS) converts CDP-DAG into PI (Figure 5, **coloured in blue)**. An unusual feature of PI is its fatty acyl composition. Nearly 80% of PI have C18:0 at the *sn-1* position and C20:4 at the *sn-2* position. This remodelling of acyl chains occurs after PI synthesis.

**Figure 6:**
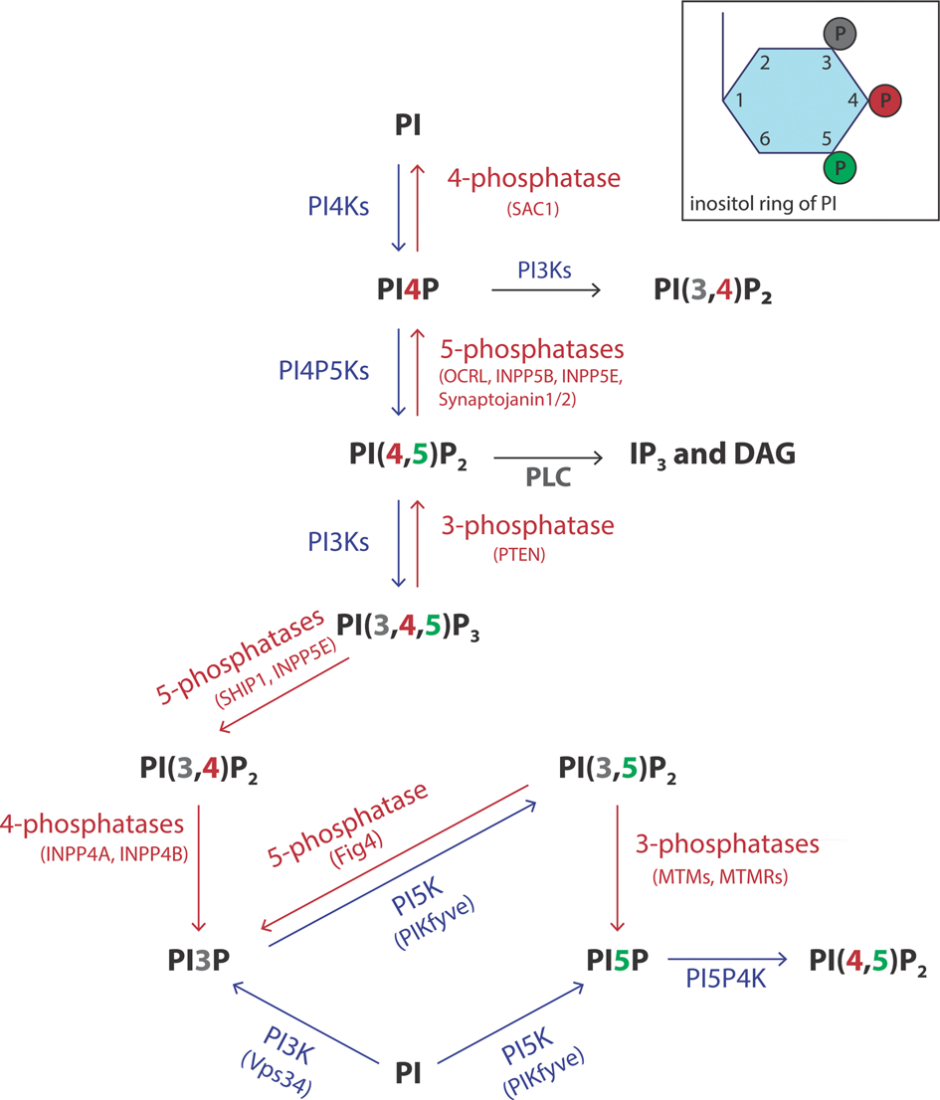
Synthesis of seven phosphorylated derivatives of PI Three hydroxyl groups of the inositol ring (positions 3, 4 and 5) of PI are accessible for phosphorylation by lipid kinases. Interconversions between various phosphoinositides occurs through lipid kinases (coloured in blue) and lipid phosphatases (coloured in red). There are multiple kinases and phosphatases with different locations in the cells. Abbreviations: INPP(5), phosphoinositide phosphatase (number at the end identifies the position of the phosphate removed); MTM, myotubularin myopathy; MTMR, MTM related; OCRL, OculoCerebroRenal syndrome of Lowe; PTEN, 3-phosphatase; SHIP, SH2 domain‐containing inositol 5′ phosphatase.

**Figure 7:**
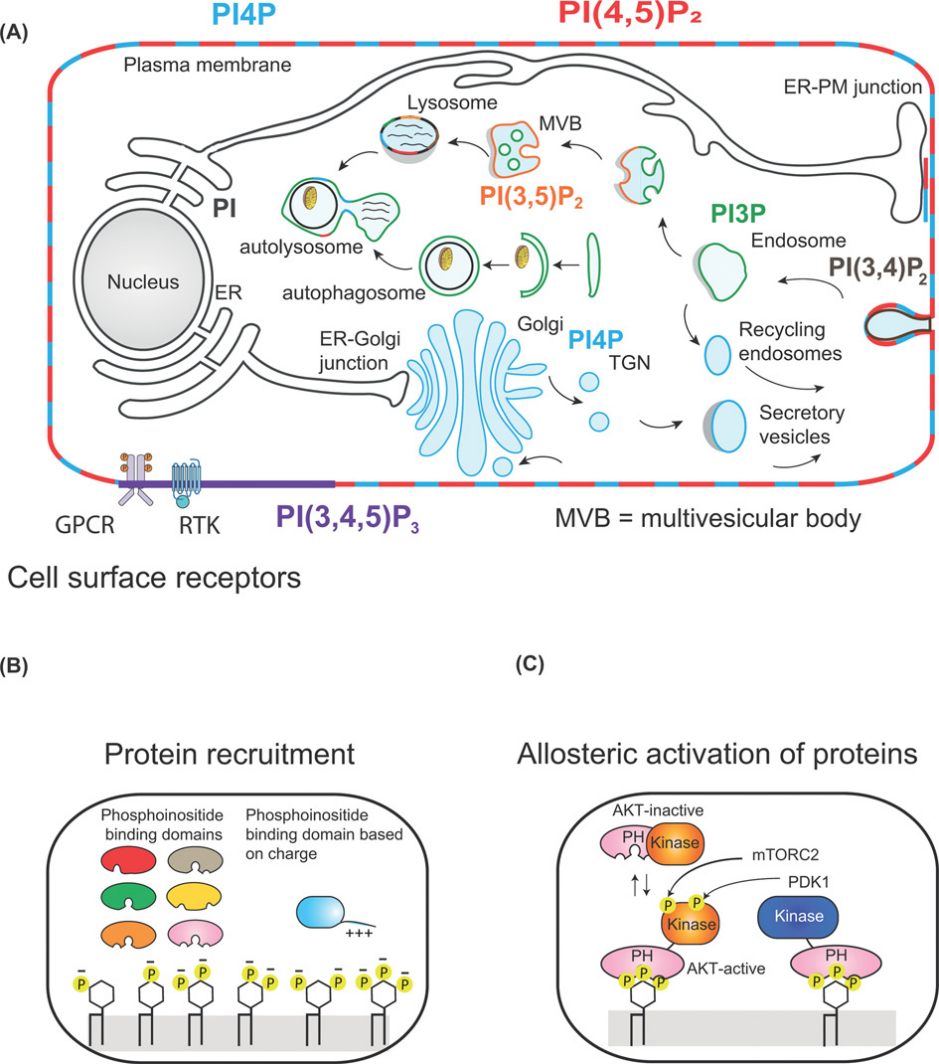
Location of different phosphoinositide species in cells (**A**) Different phosphorylated species of PI are enriched in different membrane compartments. PI(3,4,5)P_3_ (coloured purple) is only made at the plasma membrane when cells are stimulated by either GPCRs or receptor tyrosine kinases (RTKs). PI(4,5)P_2_ (coloured red) and PI4P (coloured blue) are found at the plasma membranes. PI4P is additionally present at the Golgi. PI3P is present in the endosomal compartment and PI(3,5)P_2_ is present in multivesicular bodies (MVBs) and lysosomes. (**B**) Recruitment of proteins by phosphoinositide-binding domains or by patches of positive charge present in proteins by different phosphorylated forms of PI. (**C**) Activation of AKT by phosphorylation by PDK1 and mTORC2. PIP_3_ recruits the PH domains of AKT and PDK1 to the plasma membrane bringing the two proteins together. PDK1 phosphorylates AKT. After a second phosphorylation by membrane target of rapamycin complex 2 (mTORC2), AKT is activated and can move away from the membrane to phosphorylate its target proteins (adapted from Figures 1 and 2 from Hammond G.R.V. and Burke J.E. (2020) *Current Opinion in Cell Biology*, **63**:57–67).

The inositol headgroup contains three hydroxyls at the 3, 4 and 5 positions that can be phosphorylated individually and in combination giving rise to seven different phosphorylated derivatives (Figure 6). Thus PI can be made into PI4P, PI3P, PI5P, phosphatidylinositol 4,5 bisphosphate (PI(4,5)P_2_), PI(3,5)P_2_, PI(3,4)P_2_ and phosphatidylinositol 3,4,5-trisphosphate (PI(3,4,5)P_3_). There are many lipid kinases and each enzyme can only phosphorylate one of the three available positions, 3, 4 and 5. In addition to the lipid kinases, there are phosphatases that can selectively remove the phosphates allowing for dynamic turnover (Figure 6, **kinases in blue and phosphatases in red**). Each organelle is distinguished by its phosphoinositide content and this is dependent on which lipid kinases and phosphatases are resident (Figure 7A).

### Biosynthesis of PG and CL in mitochondria

**Figure 8:**
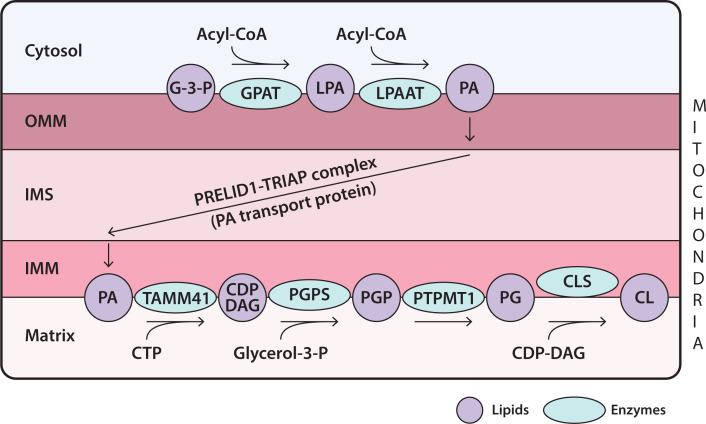
Synthesis of CL in mitochondria Synthesis of CL begins with PA which is synthesised in the OMM. It is transferred to the IMM by the PA transport protein, PRELID1–TRIAP complex. PA is translocated to the matrix side where it is then converted into PG and finally to CL through a series of enzymatic reactions all located in the IMM. See text for abbreviations of enzymes.

CL is the signature lipid of mitochondria and is synthesised exclusively there (Figure 8). CL synthesis requires PG as the intermediate. Mitochondria consist of a double membrane, the outer mitochondrial membrane (OMM) and the IMM. The space between these two membranes is the intermembrane space (IMS) and the IMM encloses the matrix compartment (Figure 8). PA is the starting point for both PG and CL synthesis. PA is synthesised in the OMM by the enzymes, GPAT and LPAAT (see Table 2). PA is delivered to the IMM by the LTP, a complex of two molecules, PRELID1 and TRIAP (Figure 8).

The enzymes for the synthesis of PG and CL are localised in the matrix side and are mainly peripheral membrane proteins with one or two exceptions. PA and CTP at the IMM are converted into CDP-DAG by the enzyme, TAMM41. TAMM41 has an identical activity with CDS enzymes present in the ER required for making PI (Figure 6). However, the enzyme bears no relationship either in amino acid sequence or in the three-dimensional structure as the evolutionary origin of the two enzymes is different. CDP-DAG and G-3-P combine into phosphatidylglycerolphosphate (PGP) catalysed by *phosphatidylglycerolphosphate synthase* (PGPS), another membrane-associated peripheral enzyme. PGP is dephosphorylated to PG by the enzyme, *protein tyrosine phosphatase mitochondrial 1* (PTPMT1). *Cardiolipin synthase* (CLS) is an integral membrane protein which catalyses the condensation of PG with another molecule of CDP-DAG to make CL. Following CL biosynthesis, the acyl chains are remodelled such that the final CL contains mainly linoleic acid (C18:2). The enzyme responsible for this transformation is *Taffazin*, a transacylase and mutations in this enzyme causes Barth syndrome, an X-linked disease. Barth syndrome is a rare condition characterised by an enlarged and weakened heart (dilated cardiomyopathy) and weakness in muscles used for movement (skeletal myopathy). This is because CL with appropriate acyl chains is crucial for mitochondrial function.

### Ether lipids and plasmalogens

**Figure 9:**
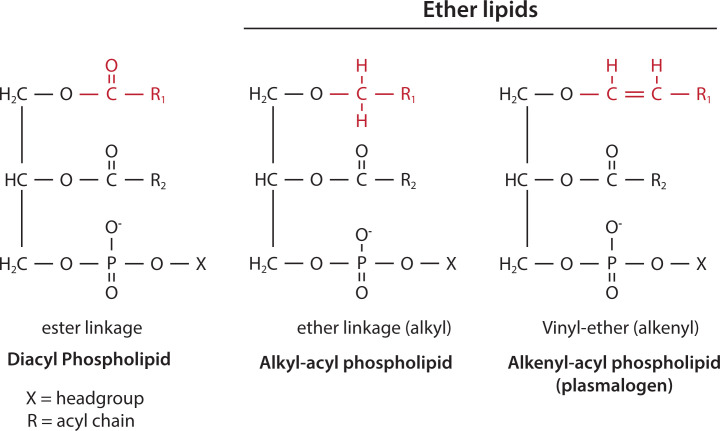
Ether-linked phospholipids Diacyl phospholipids have acyl chains linked to the *sn-1* and *sn-2* position of the glycerol backbone by ester bonds. Ether phospholipids have an acyl chain attached by an ether bond at the *sn-1* position. Ether alkenyl phospholipids have a *cis* double bond adjacent to the ether linkage at the *sn-1* position and are known as plasmalogens. The acyl chain at the *sn-2* position in ether lipids is linked via an ester linkage. The polar head group (X) of ether-linked phospholipids is mainly ethanolamine with some choline. The ester, ether (alkyl) and vinyl-ether (alkenyl) linkage at *sn-1* is coloured red.

Most phospholipids have FAs attached at the glycerol backbone using an ester bond. However, there are exceptions. A proportion of PC and PE have one of their hydrocarbon chains attached by an ether link (Figure 9) (ether-linked bonds are resistant to cleavage by phospholipases). In some tissues, as much as 20% of phospholipids are ether-linked. Ether lipids have an alkyl chain or an alkenyl chain attached to the *sn-1* position by an ether bond (Figure 9). Alkenyl ether lipids are known as plasmalogens and are mainly present as PE and some as PC. In mammals, the heart, brain tissues and inflammatory cells contain the highest concentrations of ether lipids.

Ether lipids are synthesised initially in the peroxisome and completed in the ER. After production of *sn-1*-alkyl-glycero-3-phosphate using dihydroxyacetone phosphate as the starting molecule in the peroxisome, the remaining steps occur in the ER. The alkenyl chains at the *sn-1* position consists predominantly of C16:0, C18:0 or C18:1, while the *sn-2* position is typically a polyunsaturated FA (e.g. C20:4 and C22:6). Plasmalogens are a source of arachidonic acid and DHA (C22:6 n-3) to synthesise lipid mediators such as eicosanoids, resolvins and protectins (Figure 4). Eicosanoids are derived from arachidonic acid and include prostaglandins, thromboxanes and leukotrienes. These lipid mediators are short-lived molecules that act on neighbouring cells to promote inflammation, an essential biological process for recovery from tissue injury or foreign pathogens. Acute inflammation is resolved by a different set of molecules, maresins, resolvins and protectins which are derived from DHA and EPA (Figure 4B).

Neutrophils are particularly enriched with an alkyl chain in PC. This form of PC is deacylated by an enzyme, phospholipase A_2_ (PLA_2_), to remove the FA from the *sn-2* position and the resulting lyso-PC (LPC) is reacylated with acetyl-CoA to make a potent mediator of inflammation, platelet-activating factor (PAF) (Figure 14). PAF plays a vital role in various physiological processes including mediation of normal inflammatory responses, regulation of blood circulation and pressure, regulation of coagulation responses and brain function. However, PAF is also involved in the pathogenesis of several inflammation-related chronic disorders.

## Chemical structure of sphingolipids and glycosphingolipids

**Figure 10:**
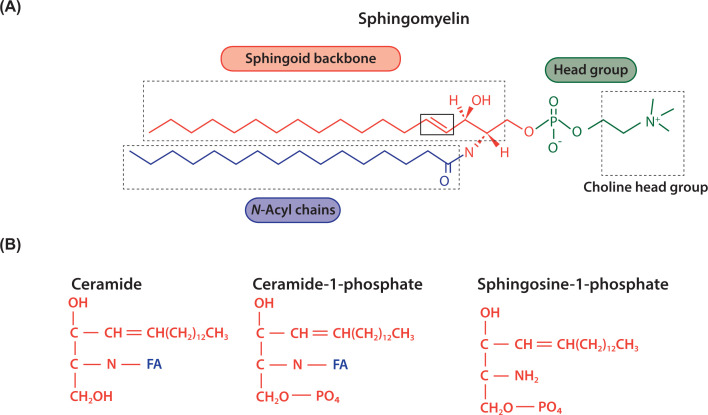
Schematic representation of sphingolipids (**A**) Sphingolipids have a sphingoid backbone (coloured red) derived from the condensation of serine with palmitoyl CoA. Attachment of an acyl chain (coloured blue) by an amide link to sphingosine makes ceramide and addition of a headgroup, phosphocholine (coloured green) makes SM, the most abundant sphingolipid of mammalian cells. (**B**) Sphingosine and ceramide can both be phosphorylated to make bioactive metabolites.

**Figure 11:**
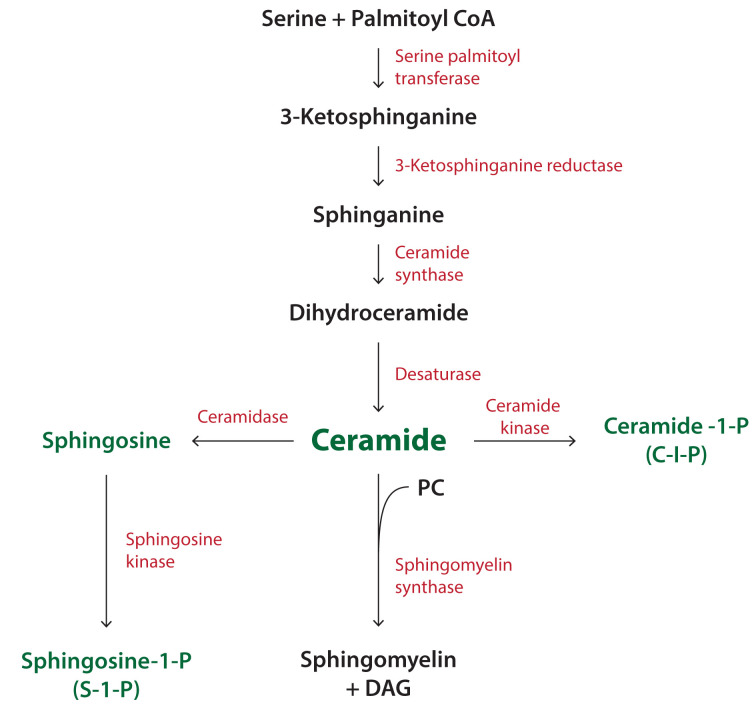
Synthesis of SM and other derivatives of ceramide Synthesis of ceramide begins with the condensation of serine with palmitoyl CoA. Ceramide can be converted into a variety of metabolites including SM, sphingosine and ceramide-1-phosphate. Sphingosine kinase phosphorylates sphingosine to make sphingosine-1-phosphate. SM can also be converted into ceramide through the action of SMase (see Figure 14). Abbreviation: SMase, sphingomyelinase.

**Figure 12:**
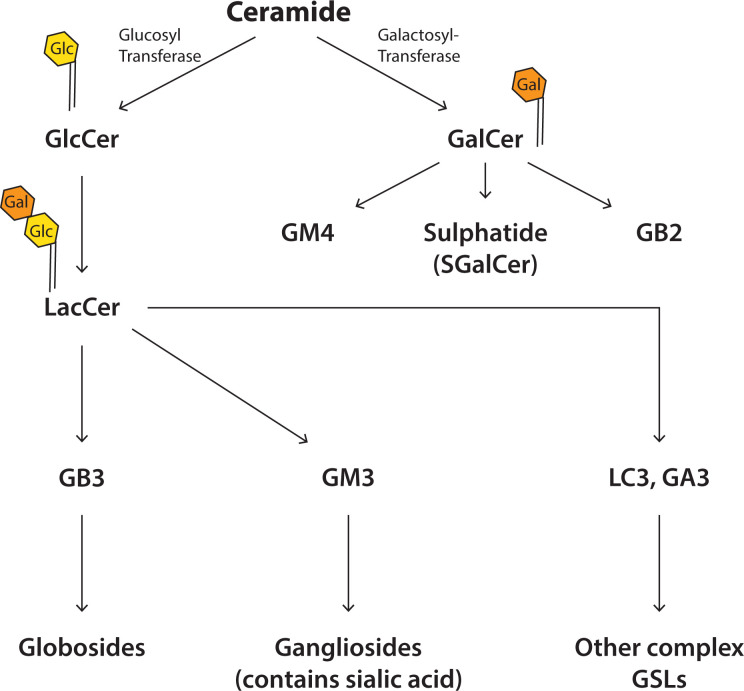
Synthesis of GSLs from ceramide Addition of the sugar molecules, glucose or galactose to ceramide makes the simple GSLs, GlcCer and GalCer. Addition of further sugar molecules generates a large number of complex GSLs. More than 400 different GSLs have been identified. The most common sugar is galactose, followed by N-acetylglucosamine and by glucose.

Sphingolipids are made from a sphingoid backbone which is made by combining palmitoyl CoA with serine (Figures 10 and 11). Attachment of an FA through an amide bond makes ceramide (Figures 10 and 11). Ceramide is the precursor lipid to make sphingomyelin (SM) and glycosphingolipids (GSLs). Addition of the phosphocholine headgroup to the hydroxyl of ceramide makes the phospholipid, SM, the most abundant sphingolipid in cells. Sugar molecules such as glucose or galactose can also be attached to ceramide and this results in the simple GSLs, glucosyl ceramide (GlcCer) and galactosyl ceramide (GalCer) (Figure 12). Addition of galactose to GluCer results in lactosylceramide (LacCer). These simple GSLs are the starting point for making a wide range of complex GSLs. Addition of sialic acid, N-acetylglucosamine, and N-acetylgalactosamine results in the production of numerous GSLs including globosides, gangliosides and sulphated sphingolipids collectively referred to as GSLs (Figure 12). The oligosaccharide chain can be very long and complex and results is an astonishing variety of GSLs, many of which have specific biological functions in cells.

### Synthesis of sphingolipids

The synthesis of sphingolipids takes place in different membrane compartments requiring the transfer of lipid intermediates by LTPs. Ceramide is synthesised at the ER via a series of reactions outlined in Figure 11. The first enzyme, *serine palmitoyl transferase* (SPT) uses serine and palmitoyl CoA to make 3-ketosphinganine. The second step is the conversion into sphinganine by the enzyme *3-ketosphinganine reductase. Ceramide synthase* (CerS) adds an acyl chain via an amide bond to sphinganine to make dihydroceramide which is converted into ceramide by *dihydroceramide desaturase.* The acyl chain composition of ceramide is determined by the specificity of the individual CerS. There are six enzymes in mammals, with each generating ceramides of a different acyl chain length. For example, CerS2 generates C22:0-ceramide and C24:1-ceramide whilst CerS5 generates C16:0-ceramide.

Ceramide has multiple fates and can be converted into SM or to GSLs (Figure 11 and 12). Ceramide is also used to make two bioactive metabolites, ceramide-1-phosphate (C-1-P) and sphingosine-1-phosphate (S-1-P) (Figure 11). Ceramide can be phosphorylated by *ceramide kinase* or it can be converted into sphingosine by the enzyme, *ceramidase*. Sphingosine is phosphorylated by *sphingosine kinases* to S-1-P. Sphingosine kinase activity is regulated by phosphorylation and by lipids, including PA.

Ceramide is converted into SM by *sphingomyelin synthase* (SMS) and this step occurs at the *trans*-Golgi (Figure 11). The movement of ceramide from the ER to the Golgi is facilitated by ceramide transfer protein, CERT. CERT tethers the ER and Golgi by interacting with both membranes to form a membrane contact site. CERT binds PI4P, a lipid that is enriched at the Golgi, through its pleckstrin homology (PH) domain (see Section, ‘PIs and their phosphorylated derivatives’). CERT also has a lipid transfer domain called StART which transfers ceramide from the ER to the Golgi. Ceramide has to be flipped from the cytosolic side to the luminal side as the catalytic site of *sphingomyelin synthase* faces the lumen of the *trans*-Golgi. The enzyme transfers the choline headgroup of PC to ceramide to make SM with DAG as a by-product (Figure 11). Since DAG levels need to be kept in check, it combines with CDP-choline to make PC catalysed by the Golgi-localised enzyme, CPT. This enzyme activity is similar to CEPT, that functions at the ER (Figure 5 and Table 2).

### Synthesis of GSLs

Glucosylceramide (GlcCer) is the precursor for most complex GSLs (Figure 12). Ceramide is converted into GlcCer at the cytosolic side of the *cis*-Golgi. Instead of using lipid transport proteins, ceramide is transferred here from the ER by vesicular traffic. GlcCer has two fates; it is used to make complex GSLs, but first it needs to be translocated to the luminal side where specific Golgi-resident glycosylation enzymes can add sugar molecules as it traverses the Golgi cisternae – from the *cis*-Golgi to the *trans*-Golgi. Alternatively, GlcCer is transported by the lipid transport protein, PI4P adapter protein 2 (FAPP2) to the* trans*-Golgi Network (TGN) directly again for conversion into a different set of GSLs by the action of TGN-resident glycosyltransferases. Again GlcCer needs to be translocated to the luminal leaflet where it is converted into LacCer by addition of a galactose molecule (Figure 12). This is an irreversible step as LacCer cannot be translocated back to the cytosolic leaflet of the cell membrane. Here the metabolism of LacCer can diverge for the formation of the different classes of complex GSLs (Figure 12). In contrast, galactosylceramide (GalCer) is synthesised at the luminal side of the ER and is transported to the Golgi complex to make other complex GSLs including GM4 and GB2. GSL complexity arises from the different number of sugar residues that can be attached and this could be as many as 20 sugar molecules. Furthermore, 12 different sugar molecules can be used with galactose being the most common.

## Biosynthesis of cholesterol

**Figure 13:**
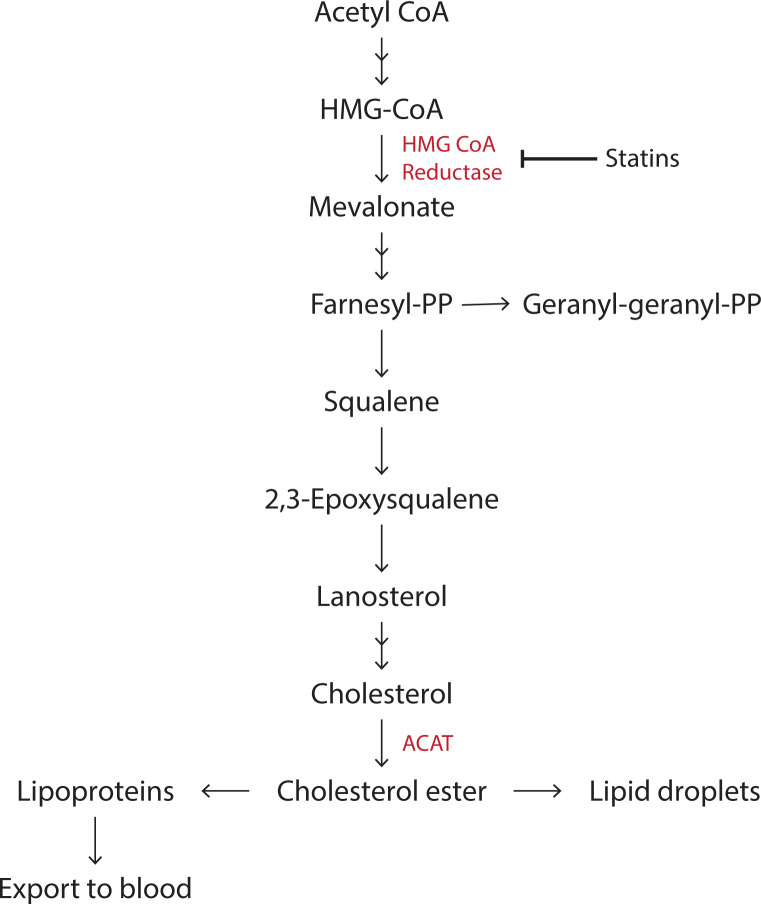
Synthesis of cholesterol and isoprenoids Cholesterol synthesis begins with acetyl CoA and the rate-limiting enzyme is HMG CoA reductase (3-Hydroxy-3-MethylGlutaryl Coenzyme A Reductase) (coloured red). Statins inhibits this enzyme. More than 20 enzymes are involved in cholesterol synthesis and only a few key steps are shown here. Cholesterol can be esterified to cholesterol ester by the enzyme, acyl CoA cholesterol acyl transferase (ACAT). The intermediate farnesyl-PP is a 15-carbon isoprenoid that can be converted into the 20-carbon Geranyl-Geranyl-PP isoprenoid. Both are used for the modification of proteins by prenylation (see Section, ‘Lipid modification of proteins’).

Cholesterol has essential roles in cells. It is important for membrane organisation and for the synthesis of steroid hormones, vitamin D and bile acids. Unlike phospholipids, cholesterol has a four-ring structure with a hydrophobic tail and a hydroxyl group (Figure 1). Addition of a FA to the hydroxyl group makes cholesterol esters usually for storage in lipid droplets (Figure 13). Most cells can synthesise cholesterol but the bulk is synthesised in the liver. In addition, cholesterol is also obtained from the diet. Enterocytes in the intestine package cholesterol into chylomicrons that are processed in the liver.

The main site of cholesterol synthesis is the ER. Synthesis starts with acetyl CoA and more than 20 enzymes are involved in the process. The key enzyme that regulates cholesterol synthesis is HMG CoA reductase, the target for statin drugs (Figure 13). The cholesterol biosynthesis pathway also provides intermediates for nonsterol isoprenoids (isoprenoid lipids are attached to C-terminal cysteines of target proteins known as prenylation to facilitate membrane attachment (Section, ‘Lipid modification of proteins’). Cholesterol biosynthesis is regulated through a system of feedback inhibition. Intracellular levels of cholesterol are sensed by sterol regulatory-binding protein (SREBP2) which is present in a complex with SREBP-cleavage-activating protein (SCAP). When cholesterol levels are high, the SREBP2–SCAP complex is retained at the ER (SCAP binds the cholesterol and results in its binding to INSIG, which retains the SCAP–SREBP in the ER). When cholesterol levels drop, SCAP binding to INSIG is reduced and the complex is transferred to the Golgi where SREBP2 is proteolytically cleaved to release its active NH_2_-terminal bHLH transcription factor domain. This enters the nucleus and activates the transcription of multiple genes involved in cholesterol synthesis and uptake.

Cells can also obtain cholesterol from the circulation. In the liver, cholesterol and TAG are packaged into very low-density lipoproteins (VLDLs) and secreted into the bloodstream. VLDL particles are processed into low-density lipoproteins (LDLs) in the circulation where the TAGs are converted into FAs. LDL contains a molecule of apolipoprotein B-100 together with many molecules of cholesterol. The LDL particle is taken into cells through the LDL receptor by endocytosis. The LDL receptor is recycled back to the plasma membrane whilst the cholesterol is transferred from the endocytic compartment via the late endosome and lysosomes to the ER. Here, cholesterol can enable down-regulation of cholesterol biosynthesis by retaining the SREBP2–SCAP complex at the ER. Mutations in the LDL receptor causes excess build-up of cholesterol in the blood (hypercholesteremia). In humans, cholesterol is deposited in the arteries leading to atherosclerosis, a major cause of heart disease.

Movement of cholesterol from the lysosome to the ER occurs through membrane contact sites. between the two organelles. Two proteins, Nieman Pick type C1 and C2 (NPC1 and NPC2), present in lysosomes, are involved. Mutations in NPC1 or NPC2 causes Niemann Pick disease, a neurodegenerative disorder, in humans. NPC2 is a small soluble protein that can bind cholesterol. It transfers the cholesterol molecule to NPC1, which is an integral membrane protein that can transport cholesterol out of the lysosome. On the cytosolic face of the lysosome, NPC1 binds to another protein, GRAMD1 that can sense the cholesterol and facilitate its removal from the lysosomal membrane. GRAMD1 possess a lipid transfer domain, called the StART domain, that allows it to shuttle the cholesterol from the lysosomal membrane to the ER. Once at the ER, this enables the shutdown of cholesterol production. A similar mechanism operates at the plasma membrane. Here, GRAMD1 connects the plasma membrane with the ER and if the cholesterol level is high at the plasma membrane, cholesterol can be sent back to the ER to shut down synthesis.

Exogenously derived or newly synthesised cholesterol that is at the ER has to be transported to the plasma membrane, where the majority of cholesterol is normally present. For this, the ORP family of lipid transporters are involved. This occurs via oxysterol-binding protein-related protein 2 (ORP2). ORPs contain a lipid transfer domain, the lipid transfer domain of ORP proteins (ORD), that can take a molecule of cholesterol from the ER and transfer it to the plasma membrane in exchange for another phospholipid, PI(4,5)P_2_.

## Phospholipid degradation by phospholipases to generate signalling metabolites

**Figure 14:**
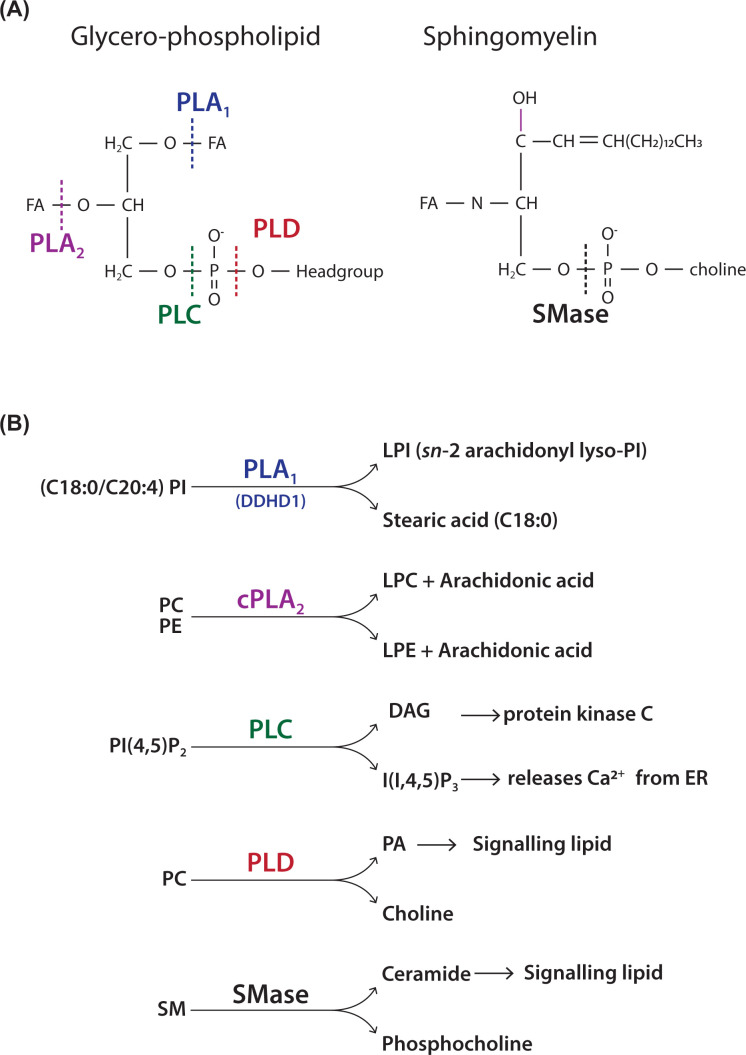
Action of phospholipases on phospholipids (**A**) Site of action of various phospholipases (A_1_, A_2_, C and D) on a typical glycerol-phospholipid. The FAs at the sn-1 and sn-2 positions are removed by phospholipases A_1_ and A_2_ respectively. Phospholipase C and D attack the phosphodiester bond at different positions. Site of action of sphingomyelinase (SMase) on SM. SMase attacks the phosphodiester bond. (**B**) Examples of well-known phospholipases that cleave specific substrates. Phospholipase A_1_ removes stearic acid from PI to make *sn-2* arachidonyl lyso-PI, a potent signalling molecule. Cytosolic phospholipase A_2_ (cPLA_2_) removes arachidonic acid from either PC or PE used for the synthesis of prostaglandins, leukotrienes and thromboxanes. Phospholipase C hydrolyses PI(4,5)P_2_ that generates two second messengers, I(1,4,5)P_3_ and DAG. Phospholipase D hydrolyses PC to make PA and choline. SM can be attacked by SMase to make ceramide and phosphocholine.

Phospholipids form the structural component of membranes. However, lipids are also a source of signalling molecules. Breakdown of specific lipids makes ‘second messengers’ that can activate intracellular processes such as proliferation of cells, secretion of hormones, muscle contraction and increase in metabolism. Enzymes called phospholipases can breakdown phospholipids and there are three different kinds of phospholipases, A, C and D (Figure 14A). Phospholipases are specific enzymes in that each phospholipase will normally attack only a single kind of phospholipid. Phospholipases present within cells are under stringent control. They only become active when cells are stimulated, e.g. by hormones, growth factors and neurotransmitters.

Phospholipases A are acyl hydrolases that remove FAs from the glycerol backbone resulting in a lyso-lipid and an FA. Phospholipases A_1_ remove FAs from the *sn*-1 position and phospholipase A_2_ from the *sn-2* position. Phospholipase B are also acyl hydrolases and can remove both FAs. Phospholipases A are a large family of enzymes and have specificity for a particular lipid. For example, for the PLA_1_ enzyme, DDHD1, the substrate is PI. Due to the specific FA composition of PI, the product of DDHD1 activity is the lyso-lipid, *sn-2*-arachidonoyl lyso-PI (LPI), an important signalling metabolite (Figure 14B). It binds to cell-surface receptors (G-protein-coupled receptor, GPCR) to activate cells. Deleterious mutations in DDHD1 causes neurological disease (hereditary spastic paraplegia), which is characterised by axonal neuropathy and gait impairments in humans. PLA_2_ enzymes are very diverse and play many important roles in cells. One major function is in the release of arachidonic acid used for making inflammatory molecules such as prostaglandins and thromboxanes (Figure 4). The enzyme responsible for this is cytosolic PLA_2_ and the main substrates are PC and PE.

Phospholipase C (PLC) and phospholipase D (PLD) both hydrolyse the phosphodiester bond of phospholipids but the cleavage site is different (Figure 14A). The main substrate for PLC is PI(4,5)P_2_, a minor phospholipid of the plasma membrane. The two products of PLC activity are second messengers; DAG which is hydrophobic and remains in the plasma membrane and inositol(1,4,5)trisphosphate (I(1,4,5)P_3_), which is water-soluble. DAG regulates the activity of a family of enzymes called protein kinase C (PKC) which can phosphorylate a number of proteins and therefore regulate their activity. Protein kinase C contains a lipid binding domain, C1 domain, that is recruited by DAG to the plasma membrane. I(1,4,5)P_3_ can diffuse to the ER where it binds to its receptor which is a Ca^2+^ channel. The lumen of the ER is a Ca^2+^ store and when the channel is open, Ca^2+^ is released in the cytoplasm. Cytosol Ca^2+^ is maintained at 100nM under resting conditions and can rise to 500 nM–1 µM. Changes in cytosol Ca^2+^ regulates many functions including muscle contraction, gene activation and metabolic regulation.

The PLC signal transduction pathway is ubiquitous and is activated by many agonists including hormones, neurotransmitters and growth factors. These bind to specific receptors at the plasma membrane of cells resulting in the activation of the PLC. Some receptors transmit their information via G-proteins and are known as GPCRs whilst receptor tyrosine kinases (RTKs) transmit their information by phosphorylation of tyrosine residues (Figure 7). GPCRs and RTKs activate different PLCs. In humans, there are 13 PLCs and are activated by different mechanisms.

PLD is also activated by GPCRs and RTKs. The substrate for PLD is the abundant membrane phospholipid, PC. The two products are water-soluble choline and the membrane-associated PA. Many functions have been attributed to PA including regulating the activity of other proteins as well as participating in membrane fusion reactions as in exocytosis (see Section, ‘The shape of the lipid regulates membrane curvature’). There are two PLD enzymes and are activated directly by monomeric G-proteins and by protein kinase C, which are downstream to GPCRs and RTKs.

SM is hydrolysed by the enzyme, sphingomyelinase (SMase) to make ceramide and choline. There are many different kinds of SMase enzymes some that are active at neutral pH and others at acidic pH. Acidic SMase localises to the lysosome where the pH is ∼5 and contributes to ceramide production. Niemann Pick diseases A and B are due to deficiencies in acidic SMase resulting in SM accumulation. Neutral SMase is activated by the cytokine, TNF-α and is part of the inflammatory response.

## Function of specific phospholipids

### PC and PE

PC and PE are the major phospholipids of membranes and they contribute to the structure of membranes but are also substrates for phospholipases. PC and PE are a major store for arachidonic acid which can released by the action of PLA_2_ enzymes. PC is also the substrate for PLD. PE is also used to make *N*-arachidonoyl ethanolamine (also known as anandamide), which is the physiological activator of the cannabinoid receptors. An acyl transferase adds arachidonic acid to the ethanolamine headgroup of PE and this is a substrate for NAPE-PLD (N-acyl PE hydrolysing PLD).

As structural lipids, the balance of PC and PE in ER membranes is important; a reduction in the PC to PE ratio leads to loss of membrane integrity due to bilayer stress. This results in activation of the Unfolded Protein Response (UPR), a gene expression programme. UPR is the stress-response pathway to maintain protein homoeostasis and is activated by either misfolded proteins or by perturbation of lipids. Lipid perturbation occurs when the PC to PE ratio is decreased. This is due to the differences in the biophysical properties of PC and PE. PC has a cylindrical shape, whereas PE is cone-shaped due to its smaller headgroup (Figure 16). The cylindrical shape is better suited for forming a bilayer unlike the cone-shaped lipid which causes membrane curvature (see Section, ‘The shape of the lipid regulates membrane curvature’ and Figure 16).

### PS

PS participates in several processes including cell signalling, endocytosis, platelet activation, blood coagulation and apoptosis (apoptosis is a complex mechanism of programmed cell death that allows the elimination of damaged cells.). In healthy cells, PS is confined to the inner bilayer of the plasma membrane. This asymmetric distribution is maintained by the flippase enzymes, the ATP-dependent aminophospholipid translocase. PS, being a negatively charged lipid, contributes to the charge of the inner leaflet of the plasma membrane. This property allows many signalling proteins that possess positively charged motifs to be attached to the plasma membranes.

Exposure of PS to the outer leaflet is a critical signal for allowing damaged cells to be removed by macrophages. Macrophages can engulf the apoptotic cells by a process of phagocytosis. It constitutes a ‘eat me’ signal when damaged cells expose their PS. Cell damage is a common occurrence and if this occurs, cells trigger self-destruction by apoptosis. Apoptotic cells translocate the PS from the inner leaflet to the outer leaflet of the plasma membrane by membrane proteins called scramblases. In apoptotic cells, proteases called caspases are activated; they inactivate the flippases and at the same time activate scramblases, irreversibly exposing the PS to the outer leaflet.

PS exposure can also usefully occur in non-apoptotic cells. In platelets, an increase in cytosol Ca^2+^ occurs after thrombin or collagen activation due to activation of PLC and I(1,4,5)P_3_ production. The increase in cytosol Ca^2+^ signals the exposure of PS by activation of the scramblase, TMEM16F. PS exposure in platelets serves as a scaffold for the activation of clotting factors for blood coagulation. Scott syndrome is a mild autosomal bleeding disorder and is caused by a deficiency in Ca^2+^-dependent PS exposure in activated platelets. This is due to mutations in TMEM16F.

### PIs and their phosphorylated derivatives

The major function of PI is to provide a substrate for making phosphorylated derivatives of PI (Figure 6). PI is also a substrate for the phospholipase A_1_ enzyme to make the signalling molecule, LPI (see Figure 14) and is also used to attach sugar molecules to make glycosyl-PI (GPI) anchors whose function will be described in Section, ‘Lipid modification of proteins’.

PI represents between 5 and 10% of the total cellular lipids. The headgroup of PI is incredibly versatile: three of the five hydroxyls can be phosphorylated resulting in seven different phosphoinositides (Figure 6). There are many kinases and phosphatases that orchestrate these conversions and this occurs in different membrane compartments. The localisation of an individual phosphoinositide provides identity to that compartment and acts as a lipid code (Figure 7).

The importance of PI(4,5)P_2_ was first established in the 1980s when it was recognised that cells, when activated by hormones, growth factors and neurotransmitters, stimulated PLC activity to make the second messengers, I(1,4,5)P_3_ and DAG (Figure 6). Subsequently, another lipid signalling pathway involving PI(4,5)P_2_ was discovered. Here the enzyme regulated by hormones, growth factors and neurotransmitters was phosphoinositide 3-kinase (abbreviated to PI3K) that makes the second messenger PI(3,4,5)P_3_ by phosphorylating PI(4,5)P_2_ (Figure 6).

In addition to the role of PI(4,5)P_2_ in cell signalling, a new paradigm involving phosphoinositides emerged. Phosphoinositides can recruit and activate cytosolic proteins to membranes. Many proteins contain lipid binding domains that can recognise a specific phosphoinositide (Figure 7B). Phospholipid binding domains differ from lipid transfer domains. Lipid transfer domains encapsulate the entire lipid and can move lipids from different membrane compartments through the aqueous medium. In contrast, phospholipid binding domains recognise the headgroup of the lipid only and binds on the membrane surface where the lipid is present (Figure 7B). These phospholipid-binding domains are generally part of a larger protein which will contain other activities such a protein kinase activity or lipid transfer domains.

The first domain to be identified was the PH domain, named after a protein called pleckstrin. There are now over 300 proteins that contain PH domains present in humans. A PH domain has a recognisable three-dimensional structure that forms the scaffold. However, it is the amino acids of the loops within the scaffold that determine headgroup recognition. Some PH domains bind with high specificity to an individual phosphoinositide such as PI(3,4,5)P_3_, PI(4,5)P_2_, PI(3,4)P_2_, PI4P and PI3P (Figure 7B). However, not all PH domains bind lipids with high affinity and specificity and some even bind proteins. Therefore, individual PH domains have to be characterised to determine both lipid specificity and affinity. In addition to PH domains, there are at least ten other phosphoinositide-binding domains now characterised. Some are, like the PH domain, a structured domain but other proteins bind phosphoinositides through a cluster of positively charged patches (Figure 7B). These include GRAM domain, FYVE and PX domains. These domains are often named after the first proteins identified.

#### Function of PI(4,5)P_2_ at the plasma membrane

Approximately only 10% of the PI is present in the phosphorylated form. The most abundant phosphorylated PIs are phosphatidylinositol-4-phosphate (PI4P) and PI(4,5)P_2_ each representing 5% each. PI(4,5)P_2_ is localised at the inner leaflet of the plasma membranes. The synthesis of PI(4,5)P_2_ is mediated by the sequential phosphorylation of PI by plasma membrane-localised PI 4-kinases and PI4P-5 kinases (Figure 6 and 7). PI(4,5)P_2_ is required for two major signal transduction pathways, PLC and PI3K, that regulate a broad spectrum of biological activities. Both enzymes are present in an auto-inhibited state; when hormones, growth factors or neurotransmitters bind to cell surface receptors, transient release in auto-inhibition takes place.

In addition to the substrate role for PI(4,5)P_2_, the intact lipid molecule is also required for a multitude of functions including exocytosis, endocytosis, phagocytosis, ion channel and transporter regulation and actin cytoskeleton remodelling. Proteins required for endocytosis, exocytosis and phagocytosis are recruited to the plasma membrane by PI(4,5)P_2_ through their lipid binding domains or by positively charged patches. As an example, during exocytosis, several components of the fusion machinery such as CAPS, Munc13-1 and synaptotagmin-1 are recruited by PI(4,5)P_2_. Syntaxin-1, another important regulator of exocytosis, is clustered by PI(4,5)P_2_ and is the site where the vesicles are docked ready for secretion. Clathrin-mediated endocytosis is also dependent on PI(4,5)P_2_. The adapter protein, AP2, that recruits clathrin, is initially recruited to the plasma membrane by PI(4,5)P_2_. For endocytosis to proceed, PI(4,5)P_2_ has to be converted into PI4P. There are several phosphatases that accomplish this including the phosphatase, OCRL (Figure 6). The name, OCRL, stands for Oculo Cerebro Renal syndrome of Lowe and mutation in this enzyme results in a human disease with defects in eyes, brain and kidney.

Many ion channels are maintained in an open state when bound to PI(4,5)P_2_. When PI(4,5)P_2_ levels decrease due to activation of PLC, ion channels close transiently, till PI(4,5)P_2_ levels are restored. Many actin-binding proteins are recruited by PI(4,5)P_2_ and can cause a change in their conformation and affect the shape of the cells. It is thought that PI(4,5)P_2_ is not homogeneously distributed at the plasma membrane but present in spatially separated clusters allowing for localised signalling to take place.

#### PI4P functions in lipid transport and in membrane traffic

The majority of PI4P is present in two compartments, the plasma membrane and the Golgi compartment. Not only is PI4P a precursor for PI(4,5)P_2_ at the plasma membrane, it is also required for importing PS and cholesterol from the ER. As described previously, both PS and cholesterol are synthesised at the ER but are mainly enriched at the plasma membrane. Transfer of PS and cholesterol to the plasma membrane occurs against a concentration gradient and therefore requires energy. The energy input is indirect as ATP is used to phosphorylate PI4P. The movement of PS and cholesterol from the ER occurs through a reciprocal exchange of PI4P by the LTPs of the ORP family (there are 11 different ORPs present in humans). At the ER, PI4P is converted back into PI by the 4-phosphatase enzyme, SAC1 (Figure 6). PI4P levels at the plasma membrane are then restored by transfer of PI from the ER by the LTPs of the phosphatidylinositol transfer protein (PITP) family and its subsequent phosphorylation by the resident PI4K.

The Golgi is enriched in PI4P and here it is an important regulator of membrane traffic and lipid biosynthesis. Both retrograde and anterograde vesicular transport of proteins from the Golgi requires PI4P. Anterograde transport refers to Golgi vesicles going to the plasma membrane or to endosomes and here PI4P controls the recruitment of the coat complex, adaptor protein -1 (AP-1) required to make the vesicles. PI4P also participates in retrograde vesicular transport where the Golgi vesicles are transported towards the early part of the Golgi and to the ER. A Golgi protein, called Golgi-phosphoprotein 3 (GOLPH3) binds to PI4P-rich Golgi membranes via its PH domain where it is required for recycling glycosylation enzymes that control GSL biosynthesis. These enzymes are packaged into vesicles containing a coat made of the coatomer complex (COPII) at the late part of the Golgi (*trans*-Golgi) and are taken back to the early part of the Golgi (*cis*-Golgi). The enzyme that makes PI4P at the Golgi is different from the PI4K residing at the plasma membrane. Interestingly, the Golgi PI4KIIIβ enzyme and GOLPH3 are both overexpressed in many cancers and are potential drug targets.

PI4P also affects sphingolipid and cholesterol metabolism mainly through the recruitment of the LTPs. FAPP2, CERT and oxysterol-binding protein (OSBP) possess PH domains that bind to the Golgi-localised PI4P. FAPP2 transfers GlcCer from the *cis*-Golgi to the TGN for GSL synthesis. CERT transfers ceramide via its StART domain from the ER to the TGN for SM synthesis. OSBP transfers cholesterol in exchange for PI4P from the ER to the Golgi. Again, the Golgi restores its PI4P levels by importing PI from the ER using the PITP family of lipid transporters and converting into PI4P.

Many RNA viruses including Sars-Cov2 hijack the Golgi-localised enzyme, PI4KIIIβ. The virus converts the Golgi into the viral replication organelle used as the platform for viral replication. The viral replication organelle needs cholesterol for its function and uses PI4P to obtain the ER-localised cholesterol using the lipid transporter, OSBP. PI4P is converted back into PI at the ER by the 4-phosphatase, SAC1. The replication organelle is replenished with more PI by another lipid transporter, a member of the PITP family where it can be phosphorylated to PI4P for another round of cholesterol transfer.

#### Signalling through PI(3,4,5)P_3_

Phosphorylation of PI(4,5)P_2_ makes PI(3,4,5)P_3_ referred here as PIP_3_. PIP_3_ production only occurs following the activation of PI3K by stimulation of cells through GPCRs and RTKs. It is made at the plasma membrane where it can recruit proteins that possess PIP_3_-selective PH domains. The most well-studied pathway downstream of PIP_3_ is the recruitment of two protein kinases, phosphoinositide-dependent kinase 1 (PDK1) and AKT (also known as protein kinase B, PKB). After recruitment of both kinases to the plasma membrane via their respective PH domains, PDK1 phosphorylates AKT; a second phosphorylation of AKT occurs through the membrane target of rapamycin 2 (mTORC2) complex (Figure 7C). These two phosphorylations activate AKT. Activated AKT no longer needs to be attached to the plasma membrane by PIP_3_ and can now depart to phosphorylate many other proteins residing in different parts of the cell. Over a hundred substrates have been identified. The targets of AKT regulate many functions including cell growth, proliferation, cell survival, and cell metabolism.

The PI3K signalling pathway is stimulated by insulin and by insulin-like growth factors. In adipocytes and in skeletal muscle, glucose uptake is dependent on PIP_3_-AKT signalling pathway and in Type II diabetes, this pathway can become resistant to insulin action. Moreover, gain-of-function mutations in PI3K are common in many cancers leading to amplified activity. In addition, loss of function mutations or loss in the 3-phosphatase enzyme, PTEN is also very common in many cancers. PTEN removes PIP_3_ by dephosphorylation. In both cases, PIP_3_ levels are higher than normal leading to uncontrolled cell proliferation. Thus, much attention is given to understanding the PI3K pathway due to its involvement in both diabetes and cancer, two diseases that affect many people globally.

#### Functions of PI(3,4)P_2_

Two separate pathways can make PI(3,4)P_2_: phosphorylation of PI4P by PI3K and by dephosphorylation of PI(3,4,5)P_3_ by 5-phosphatases (Figure 6). PI(3,4)P_2_ acts as a signalling molecule regulating cell growth, macropinocytosis, endocytosis and membrane ruffling. PI(3,4)P_2_, like PI(3,4,5)P_3_, can also recruit AKT to the plasma membrane. In addition, several PH- and PX-domain-containing proteins have been identified that can be specifically recruited by PI(3,4)P_2_. For example, during endocytosis, Snx9, a sorting nexin, is recruited via its PX domain (Figure 7A). Snx9 contains multiple domains (Snx9 is a SH3-PX-BAR domain containing scaffold protein). The BAR domain of Snx9 stabilises the highly curved membrane formed during endocytosis. Regulation of membrane ruffling and lamellipodia formation requires the reorganisation of the actin filaments and the aptly named protein, lamellipodin is required. Lamellipodin contains a PI(3,4)P_2_-specifc PH domain that mediates its recruitment to the plasma membrane.

#### PI3P in endosomal maturation and in autophagy

Endocytosis of the plasma membranes transforms the plasma membrane to an endosomal membrane and this requires the conversion of PI(3,4)P_2_ into PI3P. This gives the endosome its identity (Figure 7A). Endosomal maturation requires further accumulation of PI3P and this occurs via another PI3K (first identified in yeast and known as vacuolar sorting mutant 34 (Vps34) that phosphorylates PI to PI3P) (Figures 6 and 7) (there are three classes of PI3Ks defined by their substrates, PI, PI4P or PI(4,5)P_2_). Many proteins involved in sorting the endocytic cargo (Snx proteins), endosomal positioning (protrudin), and endosome maturation (EEA1 and Rabenosyn-5) contain PX or FYVE domains that bind PI3P. Endosomal maturation into multivesicular bodies (MVB) and finally to lysosomes requires the conversion of PI3P into PI(3,5)P_2_ by the kinase enzyme, PIKfyve. This enzyme is recruited by PI3P to the lysosome through its FYVE domain.

PI3P also plays an important role in autophagy (Figure 7). Autophagy is the degradation of the cytoplasm by lysosomal enzymes, required when nutrient levels decline. Autophagy is also required for clearance of pathogens, aggregated proteins and damaged organelles. The biogenesis of the autophagosome is orchestrated by multiple complexes containing autophagy-related proteins that first initiate formation of a pre-autophagosomal structure, termed the phagophore. The phagophore is a cup-shaped double membrane structure and emanates from the omega-some, a site on the ER enriched in PI3P and PI3P-binding proteins. PI3P-binding proteins participate in the complex sequence of signalling that results in autophagosome assembly and activity. The phagophore elongates and closes to form a mature autophagosome that will ultimately fuse with the lysosome (Figure 7). At the early stages of autophagy, PI3P-enriched membranes serve as a platform for the growing autophagosome where it recruits the proteins of the WIPI family. WIPI family proteins possess a PROPPIN domain that allows a direct interaction with PI3P.

#### Functions of PI5P

Among the seven known phosphoinositides, PI5P was the last to be discovered. It is present at very low levels and can be potentially formed by two routes: phosphorylation of PI by the enzyme PIKfyve or by dephosphorylation of PI(3,5)P_2_ by 3-phosphatases of the MTM (myotubularin) family. Dephosphorylation is thought to be the main pathway. PI5P is a substrate for the lipid kinase, PI5P4K which phosphorylates the inositol ring at the 4 position to make PI(4,5)P_2_ (Figure 6). It is unlikely that this pathway contributes significantly to PI(4,5)P_2_ production. Rather, it may be a mechanism to remove PI5P. PI5P levels are reported to increase during stress. PI5P can bind to the PHD domain of inhibitor of growth 2 (ING2), a nuclear protein. ING2 can influence the activities of various enzymes that regulate chromatin modifications. Thus, nuclear PI5P could therefore regulate gene expression.

PI5P levels also increase during infection with *Shigella flexineri*. Introduction of the virulence factor, IpgD, a PI(4,5)P_2_ 4-phosphatase into infected cells raises PI5P levels at the plasma membrane, where it recruits a protein, T-lymphoma invasion and metastasis (Tiam1), which contains a PH domain specific for PI5P. Tiam1 is a guanine nucleotide exchange factor for Rac1. Rac1 is bound with GDP in its inactive form and Tiam1 allows the exchange of GDP to GTP. Rac1 bound to GTP is active and regulates the cytoskeleton to stimulate cell motility. Tiam1 expression is increased in cancer and contributes to the spread of cancer cells to new areas of the body, a process known as metastasis.

### Functions of CL and PG

CL is a signature lipid of mitochondria, the powerhouse of cells. It is essential for maintaining mitochondrial morphology, ATP production, signalling and dynamics. Because of its distinctive shape defined by four acyl chains, rather than the two acyl chains commonly found in other phospholipids, it has unique properties (Figure 3). It can promote the formation of highly curved regions in the membrane (Figure 16). Mitochondria have a unique architecture characterised by a double membrane, the outer and IMM. The inner membrane is highly convoluted greatly increasing its surface area creating its characteristic cristae morphology (Figure 5). CL is mainly confined to the IMM where it can promote the curvature of the membrane.

The IMM houses the machinery for oxidative phosphorylation, a process central for making ATP. There are four protein complexes of the respiratory chain and CL is bound to these complexes allowing for their stable assembly. Under stress conditions, CL can be transported to the external mitochondrial membrane and this signals that the mitochondria are damaged. Mitochondria are removed by ‘mitophagy’; a process similar to autophagy by which mitochondria are engulfed and transferred to the lysosome for degradation (see Section, ‘PI3P in endosomal maturation and in autophagy’).

Mitochondria respond to cellular metabolic demand by increasing and decreasing their number by fragmentation and fusion. CL plays an important role in this process by regulating the activity of the machinery that promotes both fragmentation and fusion.

Mitochondria generate reactive oxygen species (ROS) as part of their oxidative metabolism. CL is susceptible to oxidative damage by ROS due to the presence of the unsaturated acyl chains (C18:2 n-6). Oxidised CL favours the release of cytochrome *c* into the cytosol which initiates the process of apoptosis.

PG is a major phospholipid in bacteria but in mammalian cells it is a minor component mainly as a precursor for the synthesis of CL and for BMP. In mammals, significant amounts of PG is only present in lung surfactant. Lung surfactant is produced by type II alveolar cells of the lung. It is mainly composed of PC (80%) and PG (15%). PC comprises a mixture of molecular species with dipalmitoyl-PC (C16:0/C16:0) being the dominant species. Lung surfactant is required to reduce surface tension. It occupies the air/water interface of small alveolae to prevent their collapse during breathing. Lung surfactant is initially stored in organelles called lamellar bodies, a lysosome-related organelle. These organelles contain concentric membrane layers and are secreted to release the internal membrane sheets to the extracellular space. How lamellar bodies obtain PG, a lipid synthesised in mitochondria is not known.

### BMP functions in the lysosome

BMP is a rare lipid with a unique stereochemical configuration (Figure 3) and plays key roles in endosomal/lysosomal integrity and function. It is a negatively charged lipid that is exclusively found in late endosomal and lysosomal membranes playing a crucial role in the fate of endocytosed components. It can promote lipid sorting in the late endosomes/lysosomes by activating lipid hydrolases and LTPs. Our knowledge of these rare lipid is rudimentary because of its low levels and its biosynthesis has not been fully elucidated. PG is thought to be its precursor, a lipid that is exclusively synthesised in mitochondria, most likely acquired into lysosomes through mitophagy.

The main function of BMP is in the regulation of lysosomal stability, function, enzyme activation and endosomal traffic. Late endosomes and lysosomes contain vesicles within their lumen and BMP is enriched in these intraluminal vesicles. BMP plays a crucial role in controlling the fate of other lipids including SM and cholesterol (during endocytosis, the plasma membrane which is enriched in SM and cholesterol, ends up in the lysosomes). BMP acts as a docking site and a cofactor for lysosomal enzymes such as acid SMase and lysosomal phospholipase A_2_. A build-up of SM in lysosomes is detrimental. This causes the breakdown of lysosomal membranes releasing lysosomal enzymes (e.g. cathepsin) in the cytosol which causes cell death. Acid SMase needs to bind to BMP to perform its function of hydrolysing SM to ceramide.

A link between BMP and cholesterol exit from late endosomes has also been identified. As described previously, cholesterol is transferred from the small cytosolic cholesterol transfer protein, NPC2, to the large multispanning integral protein, NPC1, to facilitate cholesterol export. NPC2 requires BMP to bind to cholesterol. BMP levels are often dysregulated in many diseases including lysosomal storage disorders, phospholipidosis (accumulation of phospholipids), metabolic diseases, liver and kidney diseases and neurodegenerative disorders.

## Sphingolipids function

### GSLs

GSLs are very diverse molecules and are involved in a variety of cell signalling events (Figure 12). GSLs are present at the external monolayer of the plasma membrane facing the extracellular medium. Their main function is to modulate the activities of many plasma membrane proteins including signalling receptors. They have far-reaching roles including immune cell function, response to viral and microbial infections and in cell development and differentiation. Lysosomal enzymes that degrade GSLs are often mutated in humans resulting in disease due to their inappropriate accumulation.

### Ceramides

Ceramides are key mediators of apoptosis, autophagy, mitophagy, cell cycle arrest and senescence. Various stress stimuli, such as tumour necrosis factor (TNFα), ionising radiation and chemotherapeutic drugs can trigger ceramide accumulation. This can occur through the stimulation of *de novo* synthesis or through activation of SMases. SM hydrolysis occurs primarily at the lysosome or at the plasma membrane. The mechanisms by which ceramides modulate signal transduction processes include activation of serine/threonine protein kinases and phosphatases. Ceramides are major regulators of cell death, mainly by promoting apoptosis and inhibition of cell growth. Ceramide with acyl chains ranging from C_18_–C_22_ are important in the skin where it forms a barrier, protecting the tissue from infection, dehydration and mechanical stress. Ceramide can also be metabolised to generate further bioactive molecules including sphingosine-1-phosphate and ceramide-1-phosphate (Figures 10 and 11).

### Sphingosine-1-phosphate and ceramide-1-phosphate

Sphingosine-1-phosphate is produced from the phosphorylation of sphingosine, a metabolite made from ceramide whilst phosphorylation of ceramide makes ceramide-1-phosphate. Both molecules can regulate a variety of physiological functions and are implicated in various pathologies. Sphingosine-1-phosphate is secreted from cells and is a circulating metabolite that is capable of triggering strong intracellular reactions through a family of five GPCRs present in several cell types and tissues. Dysregulated sphingosine-1-phosphate signalling is implicated in many diseases including cancer, the cardiovascular system, central nervous system and diabetes.

Ceramide-1-phosphate is mainly synthesised at the Golgi complex and a lipid transport protein, ceramide phosphate transfer protein (CPTP) transfers it to the plasma membrane and to other organelles. Ceramide-1-phosphate stimulates cell proliferation and migration by stimulating a variety of intracellular pathways including PI3K/AKT signalling pathway (see Section, ‘Signalling through PI(3,4,5)P_3_) and also stimulates cPLA_2_ to promote inflammation’.

## Functions of cholesterol

Cholesterol is an essential component of eukaryotic cell membranes. Cholesterol can insert into the membrane with its hydroxyl group aligned with the polar headgroup of the phospholipid. The majority of the cholesterol resides at the plasma membrane where it is in intimate contact with SM due to hydrogen bonding. It participates in maintaining plasma membrane structure, modulating fluidity and permeability. Cholesterol is also the precursor of steroid hormones, e.g. oestrogens, testosterone and of vitamin D. Steroid hormones play a central role in a range of physiological functions including reproduction and vitamin D is required for absorbing calcium from the diet. Cholesterol is also the precursor of bile acids synthesised in the liver, which participate in the intestinal absorption of fats, cholesterol and fat-soluble vitamins. Conversion of cholesterol into bile acids and the subsequent excretion into faeces provides the main mechanism for cholesterol disposal. This is important in maintaining sterol homeostasis.

Both cellular and systemic cholesterol homoeostasis is maintained by the dynamic balance of biosynthesis, storage into lipid droplets, cellular export as constituents of lipoproteins and uptake into cells. Disturbed cholesterol balance underlies cardiovascular diseases and neurodegenerative diseases.

## Storage of lipids in lipid droplets

**Figure 15:**
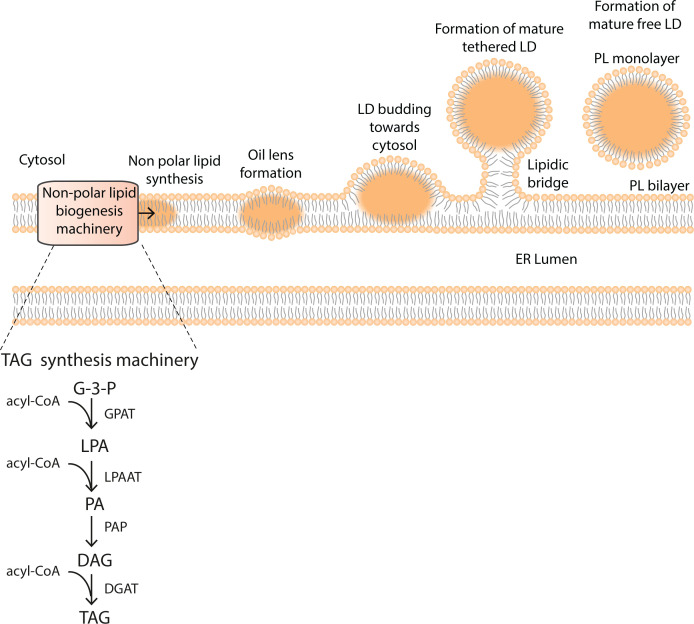
Lipid droplet formation in the ER membrane Lipid droplet biogenesis begins with the synthesis of TAG that accumulates between the two leaflets of the ER membrane. An oil lens is formed, which buds towards the cytosol. Finally, the mature lipid droplet, surrounded by a lipid monolayer, is released into the cytosol. The TAG biosynthetic machinery is recruited by a protein called seipin (not shown).

Lipid droplets are ubiquitous organelles that specialise in lipid storage. Lipid droplets are composed of a neutral lipid core surrounded by a phospholipid monolayer. The main lipids present in the core are TAGs and cholesterol esters. Lipid droplets are formed at the ER (Figure 15). The lipid, TAG is synthesised and accumulates between the bilayer forming an oil lens. The structure expands as more lipids are added till it starts to bud from the ER towards the cytosol. Several ER proteins are required including seipin, that allows TAGs to flow into the droplet. Seipin also interacts with some of the enzymes involved in triglyceride synthesis. The mature lipid droplet is linked by a lipid bridge which is ultimately severed (Figure 15). The surface of the lipid droplet is decorated with proteins such as perilipins that regulate its stability.

Lipids droplets provide a store of energy and can be mobilised by a process of lipolysis. This is a regulated by hormones. Activation of cells allows lipases to remove the FAs from TAGs and from cholesterol esters. The FAs can then be used as an energy source.

## Lipid modification of proteins

Lipids can be covalently linked to proteins to allow for their membrane attachment. This can occur after the protein has been synthesised, i.e. post-translational modification or during translation, i.e. co-translational. There are different kinds of lipid modifications that proteins can undergo and they can be reversible or irreversible. Lipid modification of proteins include acylation by FAs, prenylation or addition of glycosylphosphatidylinositol (GPI) groups. Acylation and prenylation normally occurs for intracellular proteins whilst GPI anchors are used for extracellular proteins.

FAs commonly used for protein attachment are myristic acid (C14:0) and palmitic acid (C16:0). Myristate is covalently attached via an amide bond to an N-terminal glycine residue whilst palmitate is attached via a thioester bond to a cysteine residue, often referred to as S-palmitoylation. The cysteine residues can be anywhere in the protein. The fatty acyl chain inserts into the bilayer to enable a soluble protein to be membrane-attached.

Protein palmitoylation is one of the most common lipid modifications. It is a reversible process, allowing proteins to be rapidly shuttled between membranes and cytosol in a regulated manner. Enzymes called palmitoyl transferases are responsible for the modification. These enzymes are integral membrane proteins and are distributed in different organelles and have different substrate specificities. In contrast, myristoylation occurs during protein translation. Protein myristoylation is essential for the function of proteins and target proteins include small GTP binding proteins of the ARF family and the α subunit of heterotrimeric G-proteins (proteins of the ARF family participate in vesicular membrane traffic.) It is not uncommon for proteins to be both myristoylated and palmitoylated.

Prenylation is the addition of isoprenoid lipids, another method for protein attachment to membranes and this occurs post-translationally. Prenyl chains are synthesised from acetyl CoA and share intermediates with the cholesterol synthesis pathway (Figure 13). Isoprenoids of the 15-carbon farnesyl diphosphate (FPP) and the 20-carbon geranylgeranyl diphosphate (GGPP) are built up in 5-carbon units from isopentenyl diphosphate which is synthesised downstream of mevalonate (Figure 13). Proteins that contain the CAAX motif at the carboxyl terminus can be prenylated; C is the cysteine residue where the prenyl group is attached whilst A can be any aliphatic amino acid and X denotes any amino acid. Examples of prenylated proteins are small GTP-binding proteins of the RAB and RAS family (GTPases of RAB family participate in membrane traffic whilst GTPases of RAS family are involved in activating PI3K/AKT pathway and the MAP kinase signal transduction pathways.)

Glycosyl-PI (GPI) are glycolipids that attach proteins to the extracellular leaflet of the plasma membrane. They are covalently attached to the C-termini of the proteins as a post-translational modification. The synthesis of GPI anchors is complex and begins on the cytosolic face of the ER with the attachment of N-acetylglucosamine to the 2-position of the inositol ring of the PI molecule. This molecule is then flipped to the luminal side of the ER where the synthesis is continued by addition of mannoses and ethanolamine phosphate. GPIs have a common backbone consisting of ethanolamine-phosphate, three mannoses, one glucosamine and the lipid, PI. Further addition of sugar molecules can elaborate the backbone to make a wide range of GPI anchors. The fatty acyl chains of PI are inserted in the outer leaflet of the plasma membrane. The PI molecule present in GPI anchors is 1-alkyl, 2-acyl which is an uncommon phospholipid. The acyl chain at the *sn-2* position is C18:0. The protein is attached to the GPI anchor via an amide bond between the C-terminal carboxyl group and the amino group of ethanolamine. More than 150 different human proteins are GPI-anchored and include proteins such as acetylcholinesterase, 5′-nucleotidase and adhesion molecules. GPI anchoring is essential and is required for fertilisation, development, neurogenesis and the immune system. Proteins can be released into the extracellular medium from their GPI anchors by an enzyme, GPI-specific PLD which is present in plasma.

## The shape of the lipid regulates membrane curvature

**Figure 16:**
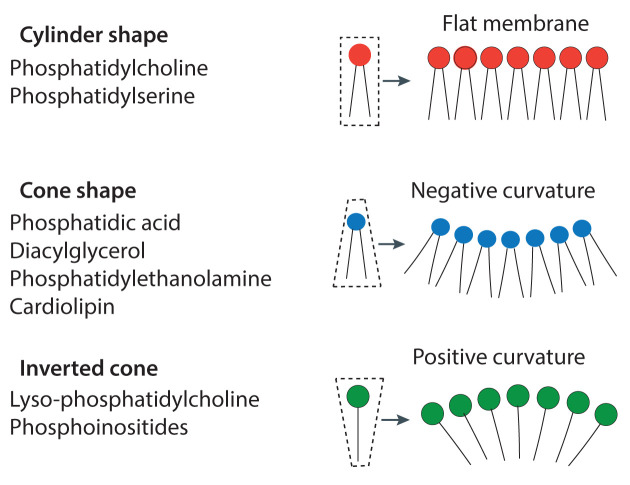
The shape of the lipid contributes to the curvature of the membrane A lipid can be: cylinder-shaped where the headgroup and the acyl chains occupy a similar-sized space, cone-shaped where the headgroup is smaller than the space occupied by the acyl chains, or inverted cone where the headgroup is larger than the space occupied the acyl chains. When present in a membrane bilayer, the lipids determine the curvature of the membrane.

The size of the headgroup of the phospholipid relative to the acyl chains influences membrane curvature. Lipids can be categorised into three shapes; cylindrical, cone shaped or inverted cone (Figure 16). This property allows membranes to adopt different curvatures. PC accounts for more than 50% of the phospholipid in most membranes and will therefore form a planar bilayer due to its cylindrical shape. Most PC molecules have a mono-unsaturated FA, which reduces the rigidity of the membrane by making it more fluid. PE assumes a conical shape because of its smaller headgroup. The presence of PE in PC bilayers will impose a curvature stress in the membrane. In mitochondria the IMM is highly convoluted and the presence of CL and PE, two cone-shaped lipids, makes it possible.

Export and import of material into cells, processes known as exocytosis and endocytosis respectively, rely on membranes to encapsulate the cargo. Cargo can be proteins, hormones and other small hydrophilic molecules. Exocytosis, endocytosis and phagocytosis, collectively referred to as vesicular traffic, requires membranes to fuse with each other or to split apart known as fission. These processes are dependent on extensive changes in lipid shapes. During membrane fusion and fission, where membrane curvature occurs transiently, increases in cone-shaped lipids such PA and DAG will facilitate negative curvature, whilst an increase in lyso-phospholipids will be permissive for positive membrane curvature (Figure 16). The dynamic turnover of lipids by phospholipid metabolising enzymes in a localised manner contributes to fusion and fission processes.

## How does one separate different kinds of lipids?

Studying the lipid composition of cells and of different organelles is challenging due to lipid diversity in both headgroup and the fatty acyl composition. Separation of lipids based on their headgroup can be achieved by using simple chromatographic procedures. This requires the extraction of lipids in non-polar solvents and one of the most common solvents used is mixtures of chloroform and methanol. The choice of the solvent depends on the nature of the lipids. Some phospholipids such as the phosphorylated forms of PI need special consideration. These lipids are highly negatively charged and therefore tend to associate with proteins. Addition of acid allows these lipids to be efficiently extracted. The extracted lipids can be separated into different classes by a variety of methods including thin layer chromatography (TLC) and mass spectrometry (MS). TLC has been the workhorse of lipid separation since the 1950s but the development of MS techniques since the 1990s is now becoming more common.

### TLC

**Figure 17:**
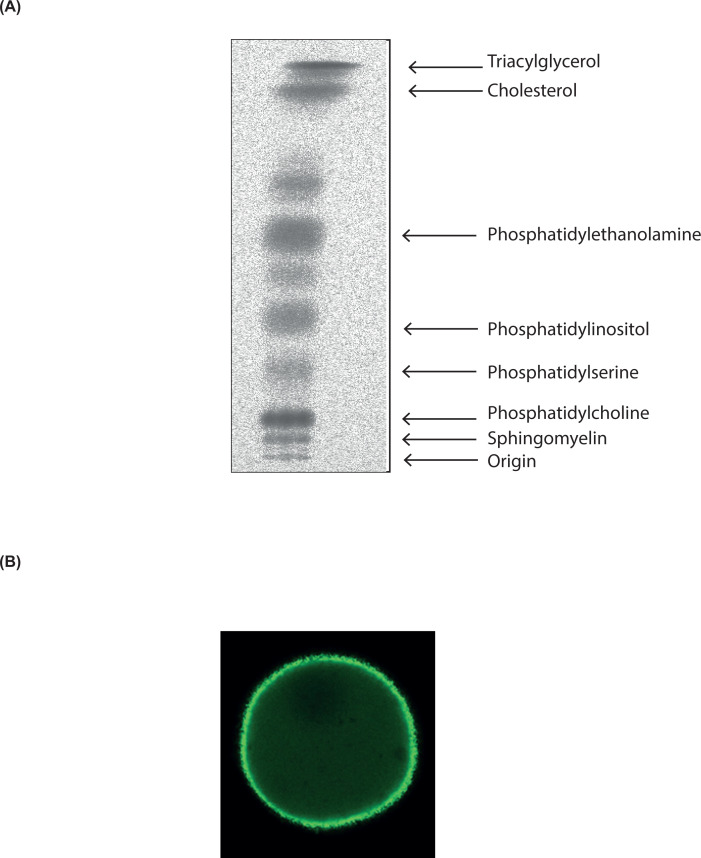
Analysis of total cellular lipids and localisation of specific lipids in cells (**A**) A thin layer chromatographic (TLC) plate showing the separation of total cellular lipids extracted from a human leukaemic cell-line, HL60 cells. HL60 cells were grown for 3 days in radioactive ^14^C-acetate which will be incorporated into all the lipids. The position of the individual lipids is indicated determined by the use of known lipid standards. Some bands are not identified. The lipids are detected by their radioactivity. The origin indicates where the mixture of lipids was applied on the TLC plate. The solvent used for separation was a mixture of chloroform:methanol:acetic acid:water (75:45:3:1). (**B**) PI(4,5)P_2_ is mainly localised at the plasma membrane. The PH domain of phospholipase Cδ1 was fused with GFP and the plasmid containing the DNA was transfected into a mast cell-line, RBL 2H3 cells. After 24 h, the cells were visualised under a fluorescent microscope. This PH domain is highly specific and will only bind to PI(4,5)P_2_.

To separate lipids by TCL, a thin layer of adsorbent such as silica or cellulose is immobilised on a sheet of glass or plastic. The lipids are applied in a single spot at one end of the sheet and the sheet placed upright in a tank with solvent. The solvent ascends the sheet by capillary action and the lipids will migrate with the solvent but with different speeds. Migration of the lipids is dependent on two factors: the solvent used to separate the lipids and the nature of the lipids themselves. Whilst this method is cheap and versatile, it will not separate all the lipids. A TLC plate is normally used to separate lipids in one-dimension allowing multiple samples to be analysed simultaneously. To obtain a higher resolution of the lipids, a TLC plate can be used to separate a single lipid sample by running it in two-dimension. The separated lipids can be detected by a variety of methods including staining with iodine vapour or dyes that bind to specific lipids. However, the most common method is to use radioisotopes to label the lipids. Cells can be labelled with radioisotopes and dynamic changes in lipids can be monitored. Radiolabelled lipids can be detected by radioautography and easily quantified (Figure 17A).

### MS-based lipidome analysis

Advances in lipid MS in the recent years has allowed a more comprehensive analyses of lipids, referred to as lipidomics. Sensitive detection of lipids is facilitated by electrospray ionisation mass spectrometry (ESI-MS) (unlike TLC, lipidomics requires expensive equipment). The molecular mass of the lipid, calculated from the mass-to-charge ratio (*m/z*) of the corresponding ion can be used for identification. Ion fragmentation, often referred to as MS/MS or tandem MS, provides further information about the molecular structure. Thus lipids can be analysed not only based on their headgroup but also by their acyl chains. Thus, the number of different lipids that can be resolved has increased and is beginning to provide new information. As an example, in cancer, it is found that PI, which normally has C18:0 at *sn*-1 and C20:4 at the *sn*-2 position in the majority of its lipids, the fatty acyl composition has shifted to shorter acyl chains and to mono-unsaturated acyl chains. Although the significance of this change is not entirely understood, it provides a fingerprint that could be utilised for diagnostics.

Lipidomics uses the principles and techniques of analytical chemistry. The most common MS-based lipidomic techniques include liquid chromatography coupled MS (LC–MS), shotgun lipidomics and MS imaging. All these different approaches have advantages and disadvantages. For example, in LC–MS, the extracted lipid sample is initially separated by high performance liquid chromatography (HPLC) prior to MS analysis and is a powerful method for targeted analysis of low abundance lipid class, but fairly slow for comprehensive analysis for cellular lipidomes. In shotgun lipidomics, the sample is directly infused into the mass spectrometer and is therefore high throughput, but minor lipids may be missed. MS imaging (MSI) is used to analyse lipids within cells and tissues and provides for the spatial distribution of lipids. Sample preparation is critical here. MS methods used for imaging lipids in tissue sections include Matrix-assisted laser desorption/ionisation (MALDI), desorption electrospray ionisation (DESI) and secondary ion MS (SIMS). These methods vary in spatial resolution, mass range and sample preparation. MALDI-MS is the most popular method. Application of MS for dynamic changes in lipid metabolism is facilitated by the use of stable isotopes such as ^13^C-labelled precursors. This technique can be used to monitor metabolic fluxes in whole organisms. ESI-MS combined with MS/MS is normally used for these kind of studies.

Lipidomics generates vast amounts of data and needs bioinformatics technology to aid in data processing for obtaining meaningful biological information. The LIPIDS MAPs website (https://www.lipidmaps.org) provides MS tools and a structural database.

## Study of specific lipids in cells

To study where lipids are localised within the cell, a different approach needs to be taken. This has been assisted by the identification of protein domains, such as the PH domain, that can bind to a specific lipid. A fluorescent probe can be made by fusing protein domains with fluorescent molecules such as green fluorescent protein (GFP). These fusion proteins can be expressed in living cells by introducing the DNA construct. The GFP-tagged protein can then be visualised using a fluorescent microscope. This is a widely used technique for the study of the phosphorylated forms of inositol lipids where many proteins domains with lipid specificity are available. An example of a cell transfected with a PH domain specific for binding to PI(4,5)P_2_ is presented in Figure 17B. The GFP fusion protein decorates the plasma membrane indicating the presence of PI(4,5)P_2_.

## Lipid organisation and composition of membranes

**Figure 18:**
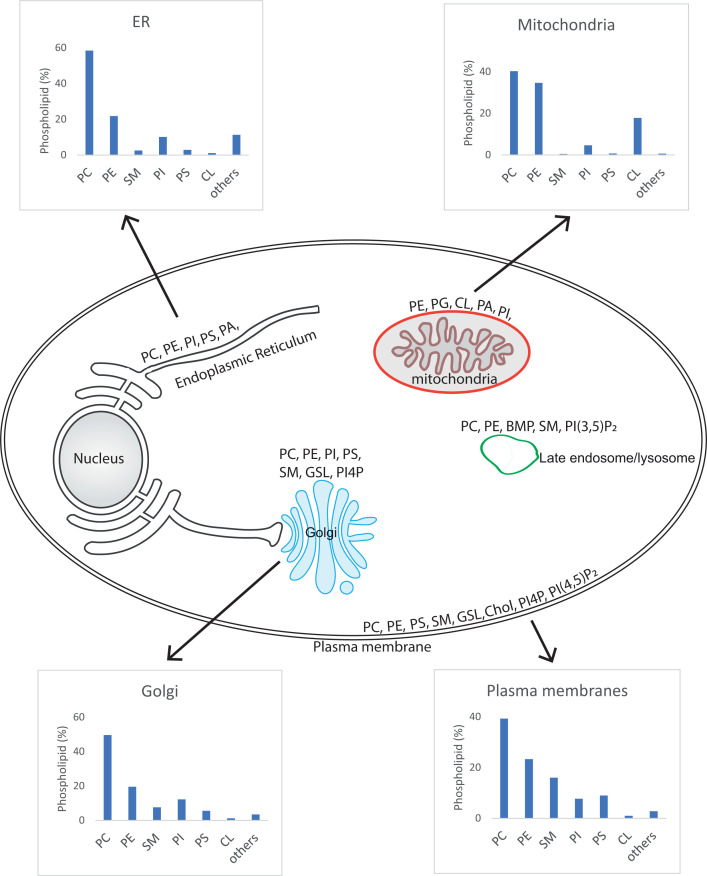
Steady-state composition of different membrane compartments of a mammalian cell The lipid composition of different membranes of the rat liver is variable. The individual graphs shows the lipid composition of the main phospholipids of a specific membrane compartment expressed as a percentage of the total phospholipid. Some minor lipids are not shown and are referred to as ‘others’. Some of the key minor lipids present in a particular compartment are included in the figure. Abbreviation: Chol, cholesterol.

The plasma membrane defines the cell boundary and intracellular membranes form the internal organelles. The phospholipid composition of these different membrane compartments are distinctive reflecting their function (Figure 18). Moreover, the two leaflets of the bilayer show asymmetric distribution of lipids. For, example, the plasma membrane comprises two monolayers, one leaflet facing toward the external environment and the other facing the cytosol. The lipid composition and the physical characteristics of these two leaflets are remarkably distinct. The red blood cell does not have internal membranes and has thus provided the opportunity to study the lipid composition of the two leaflets with great precision. The external-facing leaflet is almost exclusively composed of PC, SM and GSLs, whilst the inner leaflet is approximately equimolar at 20% between PC, PE, PS and plasmalogen-PE. Importantly, the lipids of the external-facing leaflet are fully saturated whilst the majority of the lipids facing the cytoplasmic leaflet are polyunsaturated. Both acyl chain saturation and charge of the lipid headgroup contribute to the physical and chemical properties of the membrane. Thus the outer leaflet is more rigid whilst the inner leaflet is more fluid and is also negatively charged due to the presence of PS with a smaller contribution by the phosphoinositides, PI4P and PI(4,5)P_2_.

Cell membranes also have the capacity to self organise into structurally and functionally distinct domains, conceptually referred to as the raft hypothesis. This concept arose due to the observation that cells when lysed with non-ionic detergents, yield detergent-resistant membranes that are enriched in specific lipids and proteins. The raft hypothesis is also supported by studies in synthetic membranes, where lipid mixtures separate into domains, a raft domain and a non-raft domain. In the raft domain, the lipids are more tightly packed, rich in saturated lipids, in particular, SM, GSLs and cholesterol. Importantly, it is also enriched in proteins that are lipidated. Thus, raft domains allow signalling events to be compartmentalised at the plasma membrane.

## Closing remarks

Once regarded as structural components of membranes, lipids have emerged as sources of signalling molecules to regulate many physiological functions of cells. Cellular lipids are dynamic with phospholipases, kinases and phosphatases generating new lipid species in a regulated manner. These transient changes allow for responding to the external environment to maintain cellular health. It is now clear that cell function is remarkably dependent on the right lipid environment. Different organelles of the cell are enriched in specific lipids reflecting their specialist function. The study of lipids has been the domain of biochemists till recently and this has been determined by the techniques available. With the arrival of fluorescent tags that can be added to proteins, cell biology has increased our understanding of lipid localisation and their dynamics. There are two major developments that has taken place in lipid biochemistry: the identification of lipid binding domains in proteins that allow proteins to engage with membranes in a highly regulated manner and the identification of lipid transfer domains that can exchange lipids with other membranes at membrane contact sites. New lipid-binding domains and LTPs are still being identified and is the new frontier in lipid studies. The lipid field is expanding rapidly as new techniques to analyse lipids by mass spectroscopy has given rise to lipidomics, allowing the study of the lipidome in its entirety. New lipid molecules are still being identified and we have much to learn.

## Open Access

Open access for this article was enabled by the participation of University College London in an all-inclusive *Read & Publish* pilot with Portland Press and the Biochemical Society under a transformative agreement with JISC.

## Abbreviations

BMP: bis(monoacylglycerol)phosphate
CDP-DAG: CDP-diacylglycerol
CERT: ceramide transfer protein
CL: cardiolipin
DAG: diacylglycerol
DHA: docosahexaenoic acid
EPA: eicosapentaenoic acid
ER: endoplasmic reticulum
ESI-MS: electrospray ionisation mass spectrometry
FA: fatty acid
FAPP2: PI_4_P adapter protein 2
G-3-P: glycerol-3-phosphate
GPAT: *glycerol 3-phosphate acyl-transferase*
GPCR: G-protein-coupled receptor
GSL: glycosphingolipid
IMM: inner mitochondrial membrane
LC–MS: liquid chromatography coupled mass spectrometry
LPAAT: *lyso-PA acyl transferase*
LPI: lyso-PI
LTP: lipid transfer protein
MALDI: matrix-assisted laser desorption/ionisation
MAM: mitochondria-associated membrane
MS: mass spectrometry
OMM: outer mitochondrial membrane
ORP: oxysterol-binding protein-related protein
OSBP: oxysterol-binding protein
PA: phosphatidic acid
PC: phosphatidylcholine
PE: phosphatidylethanolamine
PG: phosphatidylglycerol
PH: pleckstrin homology
PI: phosphatidylinositol
PI3K: phosphoinositide 3-kinase
PI(3,4,5,)P_3_: phosphatidylinositol 3,4,5-trisphosphate
PI(4,5)P_2_: phosphatidylinositol 4,5 bisphosphate
PITP: phosphatidylinositol transfer protein
PLC: phospholipase C
PLD: phospholipase D
PS: phosphatidylserine
ROS: reactive oxygen species
RTK: receptor tyrosine kinase
SCD1: stearoyl-CoA desaturase 1
SM: sphingomyelin
SMase: sphingomyelinase
sn: stereochemical numbering
TAG: triacylglycerol
TGN: trans-Golgi network
TLC: thin layer chromatography
